# Single-chain polybutadiene organometallic nanoparticles: an experimental and theoretical study†
†Electronic supplementary information (ESI) available: Detailed experimental procedures and characterization of ONPs including: ^1^H and ^13^C NMR, SEC, DLS, DSC, TGA, UV-vis, GC-MS spectra and computational calculations. See DOI: 10.1039/c5sc04535e



**DOI:** 10.1039/c5sc04535e

**Published:** 2016-01-07

**Authors:** Inbal Berkovich, Sudheendran Mavila, Olga Iliashevsky, Sebastian Kozuch, N. Gabriel Lemcoff

**Affiliations:** a Department of Chemistry , Ben-Gurion University of the Negev , Beer-Sheva 84105 , Israel . Email: lemcoff@bgu.ac.il; b Department of Chemical Engineering , Ben-Gurion University of the Negev , Beer-Sheva 84105 , Israel; c Lise Meitner – Minerva Center for Computational Quantum Chemistry , Israel

## Abstract

High molecular weight polybutadienes and rhodium complexes were used to produce single chain organometallic nanoparticles. Irradiation of high *cis*-polybutadiene in the presence of a photosensitizer isomerised the double bonds to produce differing *cis*/*trans* ratios within the polymer. Notably, a higher *cis* percentage of carbon–carbon double bonds within the polymer structure led to faster binding of metal ions, as well as their faster removal by competing phosphine ligands. The experimental results were supported and rationalized by DFT computations.

## Abstract 2

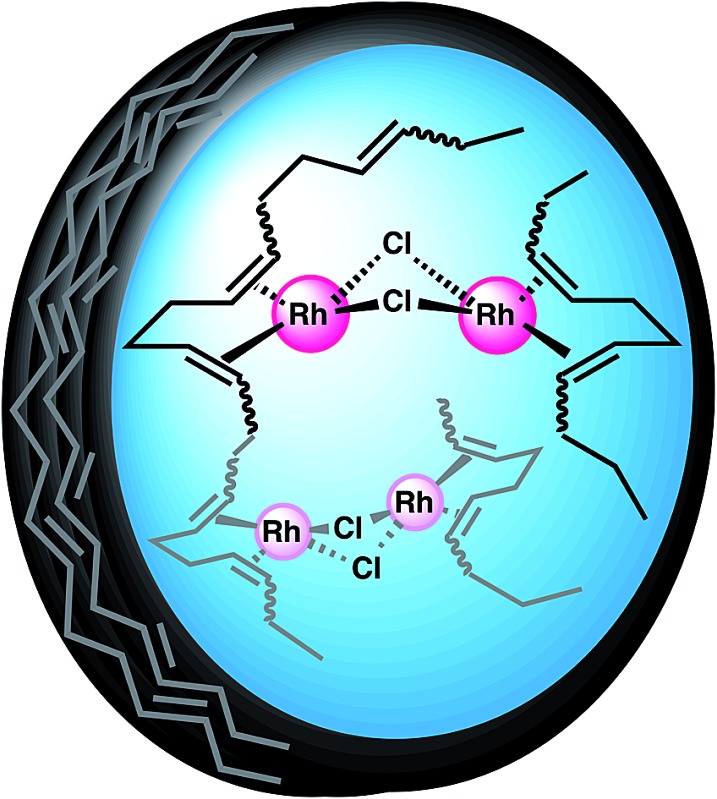
High molecular weight polybutadienes and rhodium complexes were used to produce single chain organometallic nanoparticles. A relationship was found between the *cis* double bond content of the polymer and metal binding kinetics.

## Introduction

The creation of new compounds and the study of their properties is one of the most rewarding endeavours for experimental scientists. A new area of macromolecular research deals with the study and preparation of organic nanoparticles (ONPs).^1–4^ An emerging technique for the facile generation of tailor-made well-defined ONPs is the intramolecular cross-linking of single polymer chains; these ONPs are called single chain nanoparticles (SCNPs).^5–11^ Moreover, the insertion of metals to these polymeric structures poses promising prospects for various applications, such as catalysis, drug delivery and sensing.^12–18^ In part, this potential arises because metallopolymers may combine the physical and chemical properties of both polymer and the embedded metal. Thus, the study of the factors affecting the formation, folding and architecture of metal containing SCNPs is an appealing goal.^19–25^ The concept of single chain collapse driven by metal coordination has been introduced recently by several research groups. Pomposo and co-workers reported on the preparation of organometallic SCNPs by the intramolecular cross linking of copolymers bearing β-ketoester chelating groups with Cu(ii).^19^ The use of these nanoparticles in oxidative coupling reactions revealed an enhanced catalytic specificity compared to the classical catalysts such as CuCl_2_, Cu(OAc)_2_ and Cu(acac)_2_. In another recent work, Pomposo *et al.* have extended this methodology for the preparation of water-soluble globular SCNPs with polymerase mimicking activity.^20^ Paik *et al.* detailed a cyclotetramerization cross-linking reaction within polymers bearing phthalonitrile pendant groups with CuCl resulting in SCNPs containing copper phthalocyanines.^21^ The Barner-Kowollik group recently used telechelic phosphine ligands to fold polymers with Pd(ii).^22^ Moreover, Pd-SCNPs formed by a similar strategy have shown catalytic activity for a benchmark Sonogashira coupling reaction.^23^ The carbon–carbon double bond π-electron coordination to metals is one of the most ubiquitous bonding types in organometallic chemistry.^26–28^ A recent example by Manners *et al.* shows how Pt ions may be used to reversibly cross-link a corona of polyisoprene to control the overall architecture of the macrostructure.^29^ Our group has recently developed an intramolecular chain collapse approach for the synthesis of well-defined organometallic ONPs by a direct exchange of labile ligands of chlorobis(ethylene)rhodium(i) dimer by 1,5-hexadiene units present in poly-1,5-*cis*-cyclooctadiene (PCOD) produced by ring-opening metathesis polymerization (ROMP).^24^ The method was also expanded for the preparation of SCNPs with Ir(i) and Ni(0), showing promising applications in catalysis.^25^ The polymers used were about 50 kDa in size and had 20% *cis* content; typical values for ROMP reactions using Grubbs initiators.^30,31^ While the production of PCOD by this method is fairly straightforward, we sought for a way to achieve higher molecular weight polymers and to control the *cis*/*trans* ratio, as it may widen the scope of applications for this family of SCNPs and in turn afford new insights on the intricacies of metal–π bonding in these materials. 1,4-Polybutadiene (PBD), which has the same repeat unit as PCOD, is the second largest synthetic rubber product by volume and the one with the best performance to price ratio (1.1$ kg^–1^, 18.2 performance/price ratio).^32^ In addition, it is available in high molecular weights and various *cis*/*trans* contents (depending on the catalyst used). Herein, we report on the preparation and characterization of rhodium(i) organometallic nanoparticles prepared by single chain collapse of high molecular weight polybutadienes of differing *cis*/*trans* ratios and the experimental and computational study of the effect of the double bond stereochemistry on the polymers' metal-binding properties.

## Results and discussions

### Synthesis of Rh(i)-ONPs from PBD with varying *cis* content

**Scheme 1:**
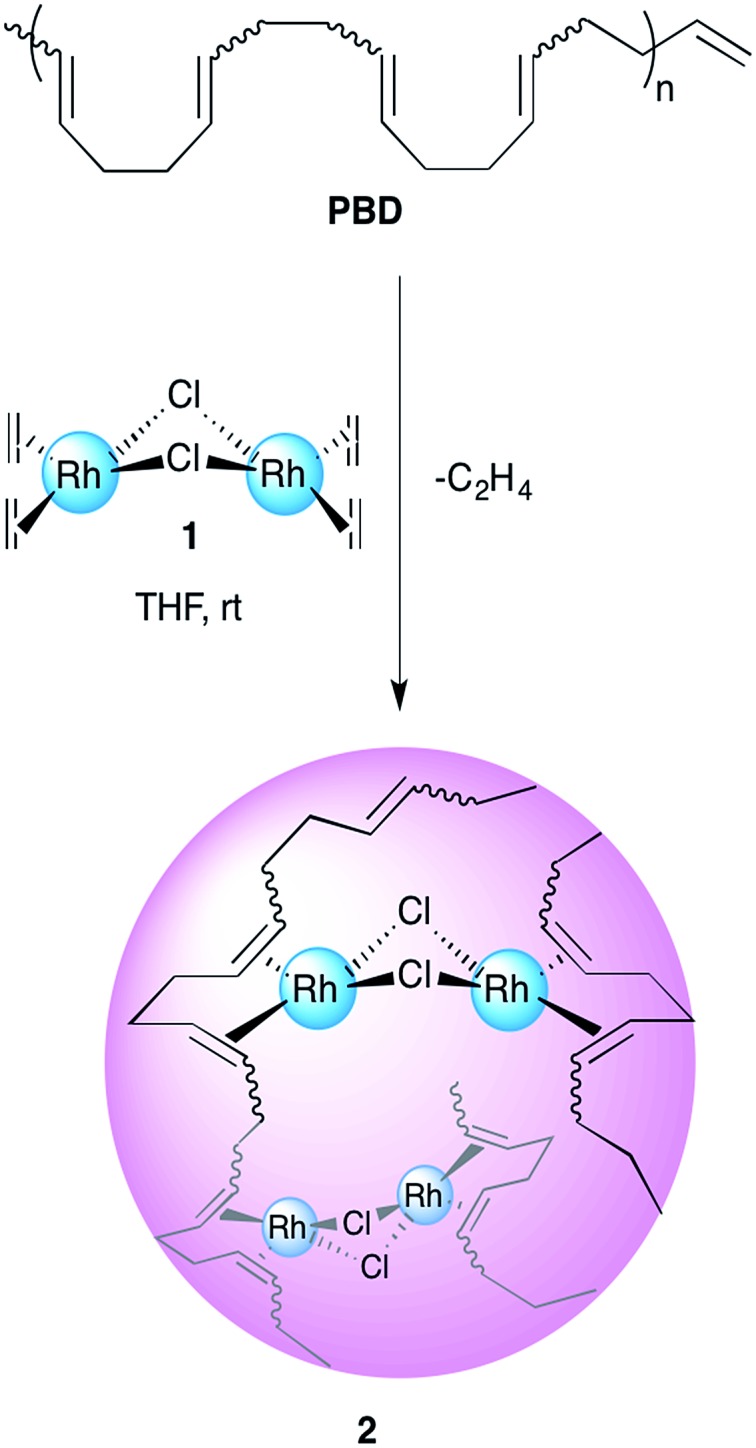
Schematic illustration of the preparation of Rh(i)-ONPs.

**Fig. 1:**
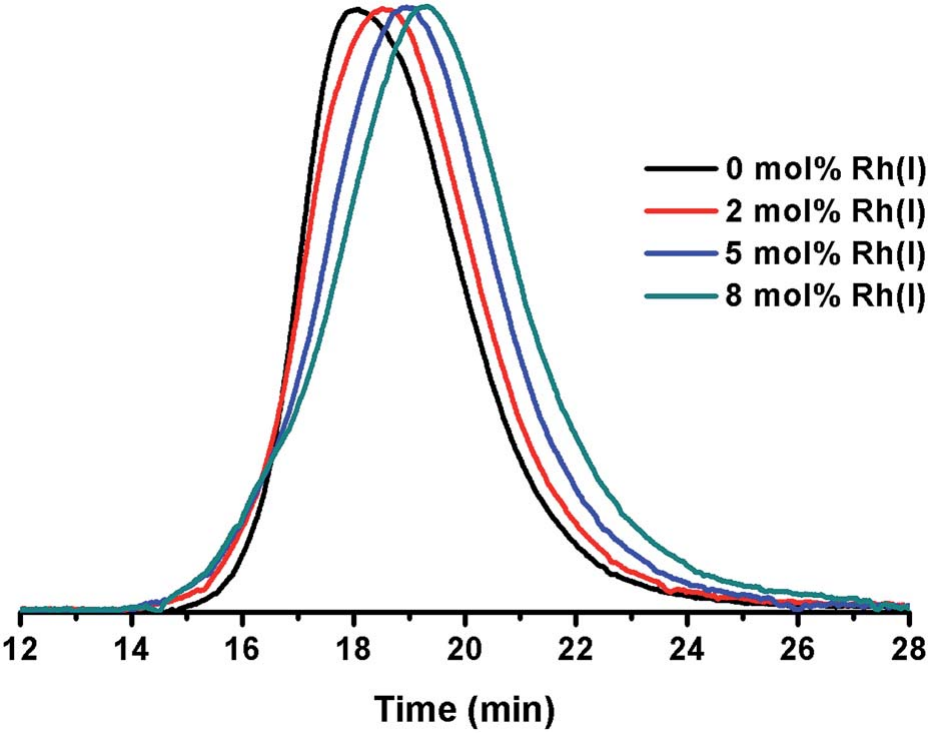
Overlay of SEC plots obtained for ONPs prepared with varying concentrations of Rh(i).

1,4-Polybutadienes with varying *cis*/*trans* content were obtained by irradiating toluene solutions of commercial PBD (3 × 10^5^ g mol^–1^, PDI = 2.5, 95% *cis*, 5% vinyl) with 365 nm UV light in the presence of diphenyl disulfide as a photosensitizer for different periods of time (Fig. S1†).^33^ The polymers obtained had a final *cis* content of 95%, 72%, 51% and 20% (determined by ^1^H-NMR) without a significant change in their molecular weights. Having obtained the series of PBDs with varying *cis*/*trans* ratios, their single chain collapse was attempted *via* the addition of chlorobis(ethylene)rhodium(i) dimer (**1**), as rhodium donor, under dilute conditions. To our satisfaction, the PBDs quickly reacted at room temperature to afford polymer bound complexes **2** (Scheme 1), as determined by UV-vis, NMR, DLS and GPC analyses. The size of the organometallic SCNP was controlled by systematically varying the percentage of rhodium(i) introduced onto the polymer chain. As in the PCOD examples, a linear dependence between the amount of rhodium(i) (cross-linker) added and the reduction in the hydrodynamic radius of the Rh(i)-ONP was observed. The degree of chain collapse was monitored by both SEC and dynamic light scattering (DLS) in THF (Fig. 1 and Table 1, Fig. S9–S12†).

**Table 1:** Rh(i)-ONPs with varying rhodium(i) content^*a*^

Entry	Rh(i) [mol%]^*b*^	*M*_w_ ^*c*^ [×10^5^ g mol^–1^]	PDI^*c*^	Intrinsic viscosity^*c*^ (mL g^–1^)	*R*_h_ ^*d*^ (nm)
1	0	3.14	2.47	168.4	27.0
2	2	3.21	1.97	150.6	23.5
3	5	2.92	1.91	110.8	16.6
4	8	3.11	1.62	85.5	12.8

^*a*^Conditions: solvent = THF, [PBD] = 0.5 mg ml^–1^ (1.5 μM), *T* = 35 °C.

^*b*^Condition: Rh(i) = [RhCl(C_2_H_4_)_2_]_2_, mol% relative to 1,5-hexadiene units.

^*c*^Condition: determined by triple-detector SEC in THF.

^*d*^Condition: determined by DLS in THF.

### Kinetic study of rhodium(i) complexation

The observation that 1,4-polybutadienes with different *cis* content readily react with **1** to produce well-defined ONPs gave us the opportunity to study the effect of the double bond stereochemistry on the metal binding propensity. Thus, the rate of rhodium complexation was monitored with stopped flow kinetics by observing the change in the characteristic UV-vis absorption spectrum.

**Fig. 2:**
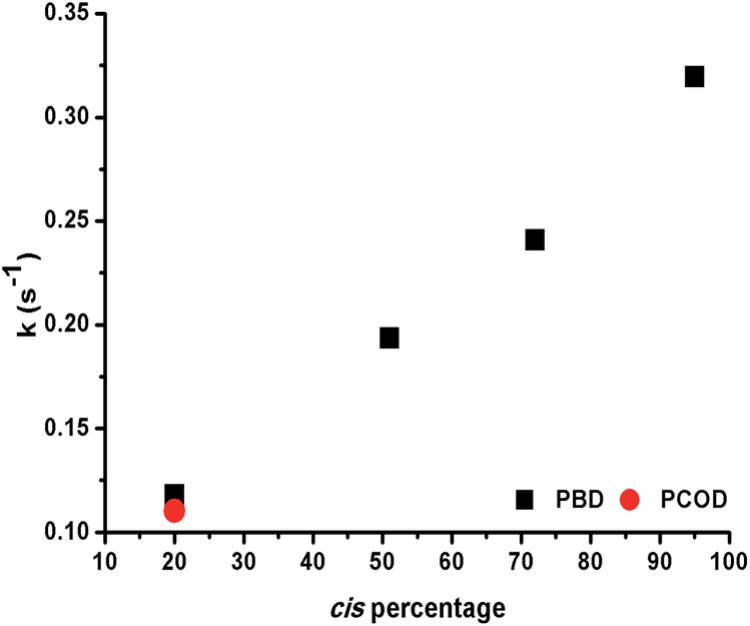
Binding rate constants *versus cis* content of PBD and PCOD for the formation of Rh(i)-ONPs.

ROMP-derived PCOD (50 kDa, PDI 1.43, 20% *cis*, 80% *trans*) was used as a control under the same conditions. In each experiment, 10 mol% of **1** was mixed with a dilute solution of polymer and a plot of the absorbance at 360 nm *versus* time was recorded. Interestingly, the rate constants increased almost linearly as the *cis* content of the polymer was raised (Fig. 2). In addition, both 20% *cis* PBD and 20% *cis* PCOD showed similar rate constants, suggesting that the rate of the complexation is independent of the polymer size or origin. The influence of the double bond stereochemistry on binding was also studied with two 4,8-dodecadienes, one all *cis* and the other 75% *trans*, as additional model compounds. The reaction of the high *cis* content 4,8-dodecadiene with **1** was about 7 times faster than the reaction of 75% *trans* 4,8-dodecadiene in accordance with the polymer experiments (Fig. S19†). These results suggest that the intrinsic stereochemistry of the carbon–carbon double bond influences the metal binding, as a similar trend was observed for both the small molecule model compounds and the polymers.

### Computational study on the effect of double bond stereochemistry on rhodium binding

Quantum mechanical computations (at the M06/def2-tzvpp(THF)//b97d3/def2-svp level,^34–38^ see the ESI†) shed some light on the reason for the faster association of the *cis* conformers of the 1,4-polybutadienes. *cis*- and *trans*-2-butene were used as models for the first step of the complexation process with complex **1**.

**Fig. 3:**
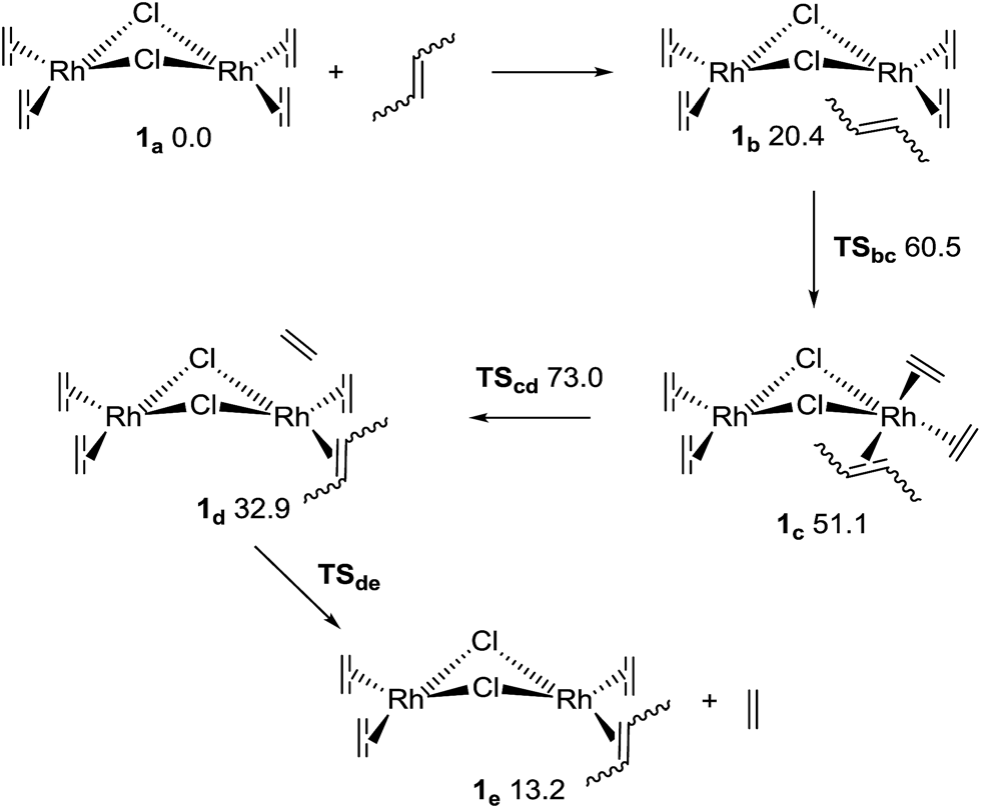
Mechanism for the first ligand substitution of 2-butene with ethene. The numbers are Gibbs relative energies in kJ mol^–1^ for the *cis*-2-butene, in a THF continuum solvent.

The first reaction with 2-butene, as shown in Fig. 3, involves four elementary steps. The first step is the formation of an adduct stabilized only by van der Waals interactions (**1_b_**), which – although exothermic – must pay a high entropy penalty, thus resulting endergonic. The second step is the insertion of the butene to form an 18e^–^ complex (**1_c_** through transition state **TS_bc_**). This is followed by the dissociation of the ethene (**TS_cd_** and **1_d_**), which results in the determining transition state for the complete ligand substitution. Finally, the ethene moiety is completely released to form **1_e_**. The complete reaction should result endergonic (at standard concentrations), but it is driven by the release of ethene as a gas. Several conformers were considered (including the inversion of the bent structure between the two coordination planes),^39^ but only the most relevant conformers will be discussed here (for higher energy structures see the ESI†). Fig. 3 shows the relative Gibbs energies in THF for the pathway including *cis*-2-butene. All the other pathways have very similar energy profiles, and therefore only the relation between the Δ*G* of the determining transition states^40–42^ (**TS_cd_**) of the various mechanisms is relevant to understand the experimentally observed kinetics for the *cis* and *trans* species. The most probable mechanism for the *trans*-2-butene substitution presents a relative Δ*G* of 76.1 kJ mol^–1^ for **TS_cd_**, only 3.1 kJ mol^–1^ higher than the equivalent state in the *cis* reaction. At room temperature this corresponds to 3.5 times faster reaction for the ethene substitution with a *cis* conformer compared to the *trans* one, agreeing with the experimental results (considering the computational and experimental differences in the reactant models).

**Fig. 4:**
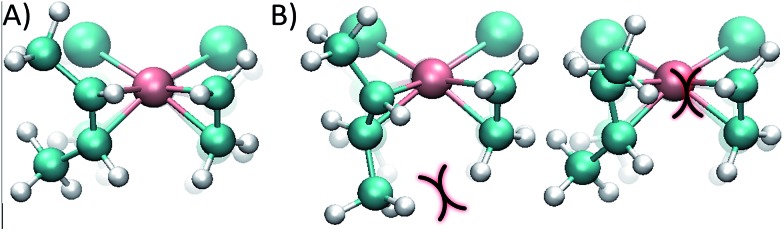
(A) *cis* conformer of the monosubstituted dirhodium complex **1_e_**, where the butene double bond is perpendicular to the complex plane, optimizing the back-bonding interaction. (B) *trans* conformers; the curves show steric impediments which diminish the π back-bonding of the metal to the ligand.

The reason for the higher energy of the *trans* pathway is shown in Fig. 4: the repulsion between the methyl group of the *trans* conformer and the ethene neighbour ligand produces a distortion of the verticality of the butene, which translates in a slightly higher energy all along the reaction (see the ESI†).

While in the *cis* conformer the Cl–Rh–C–C dihedral angle is virtually perpendicular (89°), the two possible *trans* conformers have angles of 77° and 109° (Fig. 4). Note that the chloride ligand in the opposite side of the ethene also exerts some steric hindrance, pushing out the methyl groups of the butene. However, in the *cis* case the chloride ligand pushes both methyl groups simultaneously, without affecting the dihedral angle of the butene and maintaining the back-bonding character of the metal–olefin bond.

Although the second ligand substitution may be of kinetic importance in the case of 2-butene, for chelating dienes there will be a small entropy penalty on the second substitution, as the second double bond is already in the vicinity of the metal. In the overall reaction there will be a net entropy gain when a diene species displaces two ethene molecules, and as a result the only critical step of the complete mechanism will be at the first substitution.

### Kinetic and computational study of rhodium(i) release

**Scheme 2:**
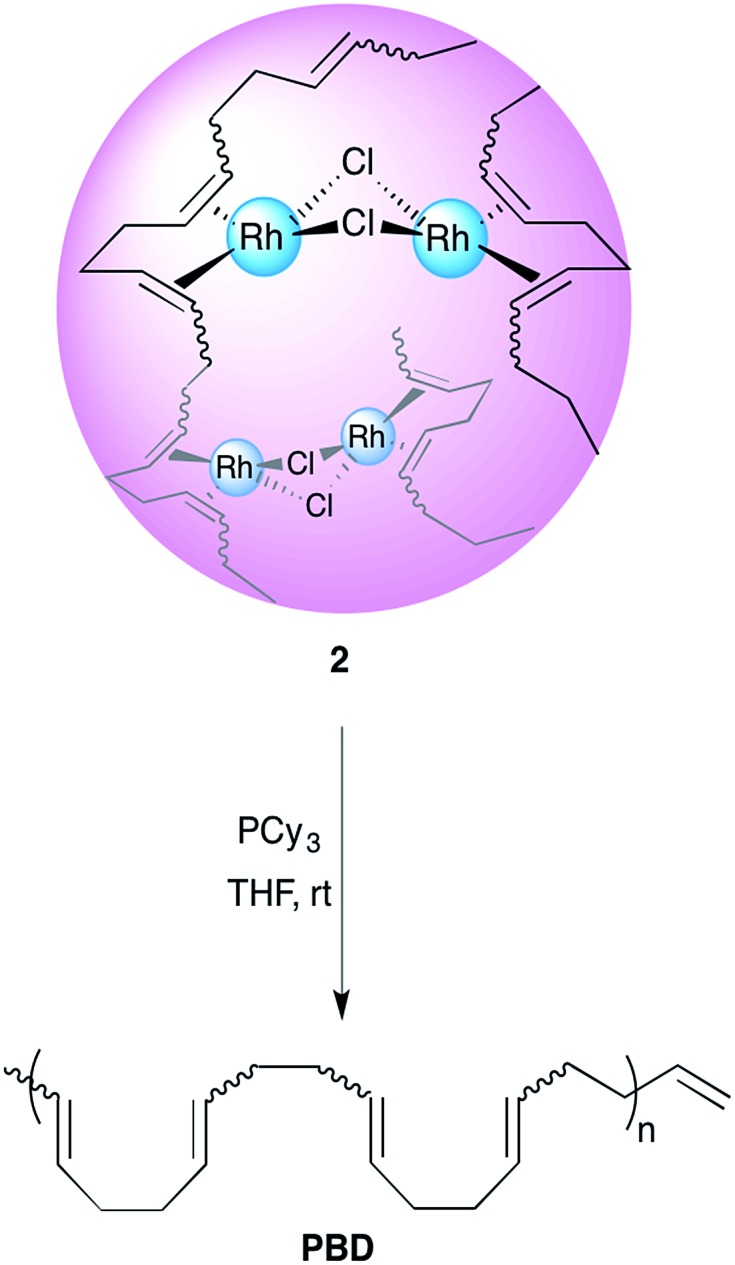
Reaction of **2** with PCy_3_.

**Fig. 5:**
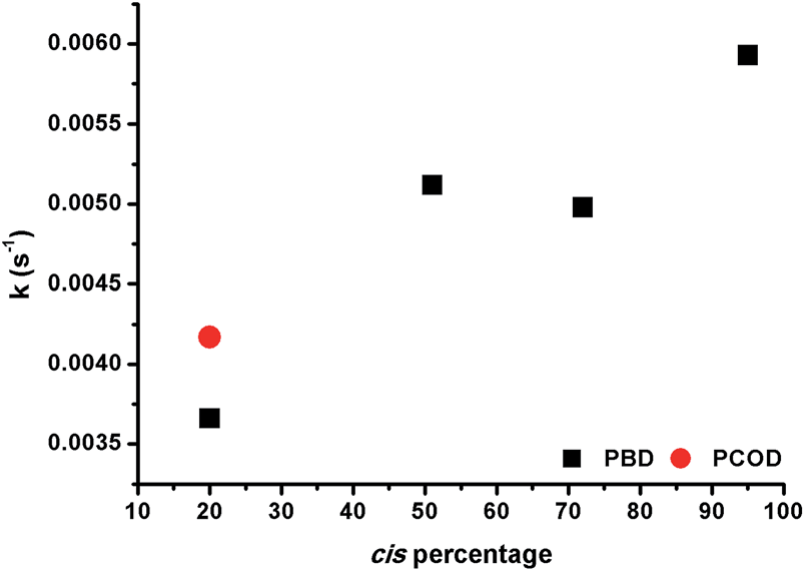
Rate constants *versus cis* content of PBD and PCOD for the disruption of Rh(i)-ONPs.

Having shown that this type of organometallic ONPs are reversible in nature,^24^ the release of Rh from PBDs was also studied by addition of a competing ligand, tricyclohexylphosphine (PCy_3_, Scheme 2). Thus, excess PCy_3_ was added to 10 mol% Rh(i)-ONPs and the absorbance at 360 nm was monitored using stopped flow kinetics. Addition of the phosphine led to a reduction in the absorption at 360 nm, indicating the disruption of the organometallic bond. A plot of the rate constants *versus* the *cis* content of the polymer showed that the reaction of 95% *cis* Rh(i)-ONPs with PCy_3_ was the fastest among the series of PBDs used; while the slowest rate constants were obtained with 20% *cis*-PCOD and PBD (Fig. 5). Also rhodium complexes of 4,8-dodecadienes were mixed with PCy_3_ and the absorption at 360 nm was monitored (Fig. S20†). The control experiment with *cis*,*cis*-4,8-dodecadiene–rhodium complex was also faster than that with the *trans* isomer.

**Fig. 6:**
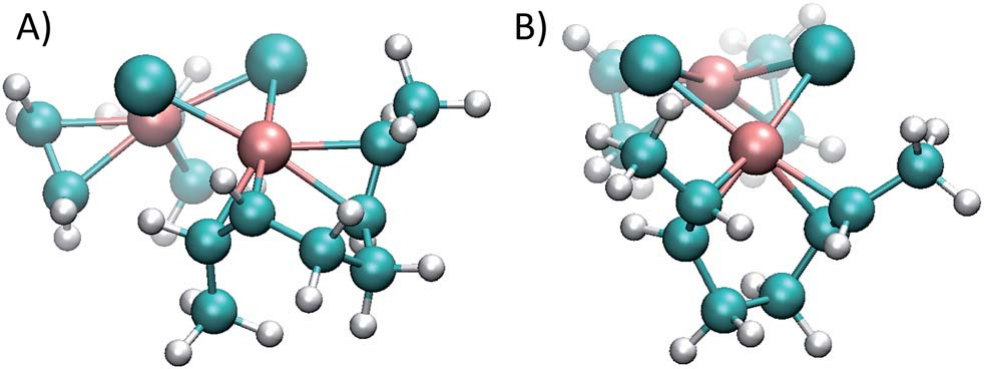
*cis* (A) and *trans* (B) 2,6-octadienes as chelating double *η* ^2^ ligands. In the *cis* species the double bonds cannot achieve the ideal vertical position that maximizes the back-bonding interaction, and therefore results in a higher energy, more reactive complex.

Thus, experiments showed that not only the association, but also the dissociation of the alkenes – driven by substitutions with PCy_3_ – are faster in the *cis* system, a fact that may seem to be at odds with the greater stability of the *η* ^2^ bond in intermediate complex **1_e_** with *cis*-2-butene compared to *trans*-2-butene. To gain a computational insight into this occurrence, 2,6-octadiene with a double *cis* or double *trans* configuration was used as a model. Computations showed that the *cis*-2,6-octadiene forms a complex 36 kJ mol^–1^ higher in energy than the double *trans* case, thus being more reactive to ligand substitution. Again, this is mainly due to strain arising when the olefin double bonds cannot achieve a vertical position (that is, perpendicular to the complex plane where the back-bonding interaction is maximized). As can be seen in Fig. 6, the double *trans* diene can be positioned with both π bonds perpendicular to the Cl–Rh–Cl plane, a preferred geometry, but the double *cis* conformer (Fig. 6A) is forced to rotate one of the double bonds.

In summary, the formation of complexes based on *cis*-dienes is faster than with the *trans* ligands because the first substitution (which determines the rate of association) has less steric impediments in the *cis* case. The dissociation of *cis*-dienes is also faster than *trans*, since the chelating diene in the *cis* conformer cannot achieve a stable geometry with both double bonds perpendicular to the complex plane, partially breaking the back bonding stabilization and making this a more reactive complex prone to a faster ligand substitution, in accordance to the experimental observations.

### The effect of the intermolecular cross-linking of PBD on the thermal properties

Having shown that dilute solutions of polymers containing the 1,5-hexadiene unit have the ability to form organometallic complexes, we sought to investigate whether the bulk polymer could also act as a metal sponge and change its properties when ions are absorbed. It has been shown that the coordination of transition metals to olefin containing polymers, such as Pd(ii) with atactic 1,2-polybutadiene, has a significant effect on the glass transition temperature (*T*_g_) of the composite.^43^ It has also been reported that Rh containing PBD copolymers (SBS) could be prepared by heating a THF or benzene solution in the presence of RhCl_3_.^44^ However, the influence of different amounts of metal coordination on the *T*_g_ of 1,4-polybutadiene has not been studied. Thus, samples of PBD were soaked overnight in THF solutions with varying concentrations of rhodium. The effect of this procedure was studied by analyzing the change in the glass transition temperature of the polymer sample and the shift in the UV-vis absorption spectrum (Fig. S21†). Indeed, as shown in Table 2, the metal led to a significant change in the polymer's physical properties as expected from the intermolecular cross-linking caused by the metal bridges.

**Table 2:** *T*_g_ of 95% *cis*-PBD with varying amount of Rh(i)

Entry	Rh(i)^*a*^ [mol%]	*T*_g_ (°C)
1	0	–101.9
2	5	–88.5
3	10	–71.2

^*a*^mol% relative to 1,5-hexadiene units.

## Conclusions

Polybutadiene, an abundant starting material, was used for the first time as the precursor single polymer chain for the formation of organometallic SCNPs. Because these polymers are much larger than those previously used, they may bind many more metal ions per nanoparticle; an important requisite for future applications (*e.g.* templates for inorganic nanoparticles). Interestingly, the introduction of rhodium ions to neat PBD produced increased glass transition temperatures as a consequence of the restricted local chain mobility caused by the metal bridges. By investigating polymers with differing *cis*/*trans* ratios, we have experimentally and computationally shown that there is a stark difference in the rate of metal sequestration and release when polymers with different microstructures are used to form organometallic nanoparticles. Indeed, polymers with higher ratio of *cis* double bonds bind rhodium ions faster and, when competitive ligands are added, also release them faster. To put this in perspective, under our experimental conditions the rhodium ions could be bound in less than ten seconds by a 300 kDa all *cis*-PBD and they could be completely removed in about seven minutes by the addition of excess phosphine, while with a high *trans*-PBD it would take thrice as long. The availability of *cis*-selective olefin metathesis catalysts^45,46^ may allow the formation of novel 1,5-cyclooctadiene polymers or copolymers with high *cis*/*trans* ratios which may be useful for quick binding and release of selected metal ions for future applications of this method. DFT based computational studies supported the experimental results and revealed the mechanistic and steric reasons for the behaviour observed. Taking into account the great importance of PBD cross-linking in the rubber industry, the study of soft, reversible, cross-linking methodologies by metals and the way the polymer structure affects the metal intake is of significant value.
