# Supplementary material for: Single-chain polybutadiene organometallic nanoparticles: an experimental and theoretical study
Source: Chem Sci. 2016 Jan 7;7(3):1773–8. doi: 10.1039/c5sc04535e (PMC5592374; doi:10.1039/c5sc04535e)
Supplement: Supplementary file 1 [file SC-007-C5SC04535E-s001.pdf]

Electronic Supplementary Material (ESI) for Chemical Science.
This journal is © The Royal Society of Chemistry 2016

Supporting Information

Single-chain polybutadiene organometallic nanoparticles: An experimental and theoretical study

Inbal Berkovich,[a] Sudheendran Mavila,[a] Olga Iliashevsky,[b] Sebastian Kozuch[a,c] and N. G. Lemcoff[a]*

[a] Department of Chemistry, Ben-Gurion University of the Negev, Beer-Sheva 84105, Israel

[b] Department of Chemical Engineering, Ben-Gurion University of the Negev, Beer-Sheva 84105, Israel

[c] Lise Meitner - Minerva Center for Computational Quantum Chemistry

Email: Lemcoff@bgu.ac.il

Table of Contents

1. Experimental 2-5
2. NMR data 6-9
3. SEC and DLS data 10-13
4. UV and stopped flow data 14-19
5. GC-MS chromatograms 20-21
6. DSC and TGA data 22-24
7. Computational section 25-39
8. References 40

Experimental

General Remarks. All manipulations were performed under dinitrogen atmosphere in a glove box (LabMaster 130, MBraun Garching, Germany). All commercially available solvents and reagents were of reagent grade quality and used without further purification except for THF, which was dried over sodium/benzophenone and freshly distilled and Diphenyl disulfide that was recrystallized from MeOH. The Rh-based complex $[RhCl(C_2H_4)_2]_2$ (**1**) was obtained from Strem chemicals. Polybutadiene (98% *cis*, average M_w 200,000-300,000 gmol^{-1}) and 2nd generation Grubbs' catalyst were obtained from Aldrich Chemical Co. Israel. Polybutadiene was purified by dissolution in toluene and filtration of insoluble material, followed by dissolution in toluene containing 0.05% BHT. Polycyclooctadiene (PCOD) was synthesized according to literature procedure using 2nd generation Grubbs' catalyst.[1] All nuclear magnetic resonance (NMR) spectra were acquired on a Bruker Avance DMX400 or DMX500 spectrometer, chemical shifts, given in ppm are relative to Me_4Si as the internal standard, or using the residual solvent peak. GC-MS analyses were done in an Agilent 6850 GC-MS apparatus with Agilent Mass Selective Detector G25577A and Agilent 19091S-443E HP-5MS 5% Phenyl Methyl Siloxane column after dissolution of the compounds in organic solvents. Irradiation was carried out using a Rayonet RPR-200 instrument with 350 nm lamps. Molecular weights and polydispersity indices (PDIs) of the polymers were determined by size exclusion chromatography (SEC) analyses in tetrahydrofuran (THF) at 35 °C using an Agilent 1200 HPLC equipped with two Agilent PLgel 5 μm Mixed-C columns and a Phenomenex Phenogel 5u 103A column. The flow rate was set to 1 mLmin^{-1}. Wyatt's miniDAWN TriStar laser light-scattering system, ViscoStarTM viscometer, and OptilabVR rEX refractometer were used as detectors. Prior to measurements, polymer solutions were filtered through Millipore 0.22 μm filters. Reaction kinetics was determined using Photophysics SX20 stopped flow instrument at room temperature. Dynamic light scattering (DLS) spectra were collected by using CGS-3 (ALV, Langen, Germany). The laser power was 20 mW at the He-Ne laser line (632.8 nm). Correlograms were calculated by ALV/SLE 5003 correlator, which were collected at 90°, during 10 s for 20 times, at 25 °C. The correlograms were fitted with version of the program CONTIN (provencher, 1982). Differential scanning calorimetry (DSC) was obtained using a METTLER TOLEDO DSC 823^e and results were evaluated using the STARe software. All experiments were performed with nitrogen flow rate of 80 mL/min at a heating rate of 10 ^{o}C/min. Each sample was subjected to 1 heating-cooling cycle. The sample containing 5%

Rh(I) was heated at 40 ^{o}C/min due to difficulty in observing the T_g and it was subjected to 2 heating-cooling cycles. Thermal gravimetric analysis (TGA) was obtained using a METLLER TOLEDO TGA/SDTA851^e. All experiments were performed with nitrogen flow rate of 50 mL/min at a heating rate of 10 ^{o}C/min. Each sample was heated at 25-1000 ^{o}C and the results were evaluated using STARe software.

General procedure for the preparation of PBD with varying *cis* content.[2] 95% *cis*-PBD (240 mg, 0.8 μmol) was transferred to a 50 ml RB flask and dissolved in 40 ml toluene for 16 h. Diphenyl disulfide (120 mg, 0.55 mmol) was added and the flask was purged with N_2 for 30 min. The flask was immersed in a water bath in order to maintain the temperature below 30 °C and then irradiated at 350 nm for 2.5, 5, and 30 min to obtain 72%, 51% and 20% *cis* content polymers, respectively. The reaction was quenched by the addition of BHT (1-2 g) and precipitated in excess of MeOH. The precipitate was then washed with MeOH and dissolved in 10 ml toluene containing 0.05% BHT as a preservative. The solvent was evaporated and dried *in vacuo* to afford PBD (208 mg, 87%). ^{1}H-NMR (400 MHz, $CDCl_3$, ppm): δ = 5.44-5.37 (m, 4H), 2.08-2.03 (m, 2H): 5.44-5.39 (-CH=CH-, *trans*), 5.39-5.35 (-CH=CH-, *cis*), 2.12-2.05 ($-CH_2-CH_2-$, *cis*), 2.05-1.99 ($-CH_2-CH_2-$, *trans*)

Procedure for the preparation of organometallic nanoparticles of rhodium(I) (2). An NMR tube was charged with 95% *cis*-PBD (4.7 mg, 0.015 μmol) dissolved in 0.4 ml of CD_2Cl_2. A solution of **1** (0.2 mg, 0.51 mmol) in 0.2 ml of CD_2Cl_2 was added to the PBD solution in one portion. The mixture was mixed well and kept overnight at room temperature. ^{1}H-NMR (400 MHz, CD_2Cl_2, ppm): δ= 5.42-5.35 (bm, 2H, -CH=CH-), 4.45-4.15 (bm, Rh(-CH=CH-), 2.09-2.06 (bm, 4H, $-CH_2-$).

General synthesis of Rh(I)-ONPs with varying percentage of Rh(I). In a glove box, 20 mg (0.06 μmol) of the PBD (average M_w = 350,000 gmol^{-1}, PDI = 2.1) was dissolved in dry THF (30 mL) in a 50 mL RB flask. A 2.1 mM stock solution of $[RhCl(C_2H_4)_2]_2$ was prepared by dissolving 4.2 mg (0.01 mmol) in 5 mL of dry THF. 5 mL of the PBD stock solutions (2.5 mg PBD, 7.1 nmol) were withdrawn into three 20 mL drum vials. A 2.0 mM stock solution of $[RhCl(C_2H_4)_2]_2$ (2%, 110 μL, 0.23 μmol), (5%, 280 μL, 0.58 μmol) and (10%, 450 μL, 0.94 μmol) were added drop-wise to each 5 mL polymer solutions under gentle stirring. The reaction mixture was stirred for 16 h at room temperature to afford Rh(I)-ONPs in THF.

Kinetic study of rhodium complexation to PBD with varying *cis/trans* ratios by stopped flow. 5 ml of each PBD/PCOD solution (0.5 mg/ml in THF) and **1** (0.3 mM in THF) were

introduced separately into 5 ml syringes. The formation of Rh(I)-ONPs was monitored at 360 nm. Rate constants were fitted to a single exponent.

Kinetic study of ligand exchange of Rh(I)-ONPs with PCy_3. Rh(I)-ONPs (10 mol% Rh(I)) were prepared from each PBD/PCOD (1.4 μM in THF) according to the synthetic procedure described above. Then, 5 ml of each Rh(I)-ONPs solution and PCy_3 (0.014 M in THF) were introduced separately into 5 ml syringes. The decrease in the Rh-olefin absorption peak was monitored at 360 nm. Rate constants were fitted to a single exponent.

Preparation of *cis,cis*-4,8-dodecadiene.[3] Potassium *tert*-butoxide (0.7 g, 6.25 mmol) was added to a suspension of butane-1,4-diylbis(triphenylphosphonium) bromide (2.0 g, 2.70 mmol) in diethyl ether (40 mL) at 0 °C. The yellow suspension was stirred at 0 °C for 30 min. Butyraldehyde (0.6 mL, 6.67 mmol) was added drop-wise at 0 °C. The resulting mixture was stirred at room temperature for 60 h. The reaction mixture was quenched by the addition of water (5 mL) and diluted with hexane (50 mL). The organic phase was separated, washed with water (30 mL), brine (20 mL) and dried over anhydrous $MgSO_4$, and then the solvent was evaporated *in vacuo*. The crude product was purified by silica-gel column chromatography using hexane as eluent to afford *cis,cis*-4,8-dodecadiene (50 mg, 11%) as colourless oil. ^{1}H-NMR (500 MHz, $CDCl_3$, ppm): δ = 5.42-5.35 (m, 4H), 2.08-1.99 (m, 8H), 1.41-1.33 (m, 4H), 0.92-0.89 (m, 6H). ^{13}C-NMR (125 MHz, $CDCl_3$, ppm): δ = 130.3 (-CH=CH- *cis,cis*), 129.5 (-CH=CH- *cis,cis*), 29.5 (-CH_2-, *cis,cis*), 27.6 (-CH_2-, *cis,cis*), 23.0 (-CH_2-CH_3-, *cis,cis*), 13.9 (-CH_3, *cis,cis*). GC-MS (EI): m/z M^+ calculated 166.17, found 166.00.

***cis-trans* isomerization of *cis,cis*-4,8-dodecadiene.**[2] Diphenyl disulfide (9.7 mg, 0.04 mmol) was added to a solution of *cis,cis*-4,8-dodecadiene (50.0 mg, 0.30 mmol) in 3 ml hexane. The solution was purged with Ar and irradiated at 350 nm for 1 hour. The product was purified by silica-gel column chromatography using hexane as eluent to afford 4,8-dodecadiene (~75% *trans*) (31 mg, 62%) as colourless oil. ^{1}H-NMR (400 MHz, $CDCl_3$, ppm): δ 5.43-5.32 (m, 4H), 2.08-1.93 (m, 8H), 1.40-1.31 (m, 4H), 0.92-0.86 (m, 6H). ^{13}C-NMR (100 MHz, $CDCl_3$, ppm): δ 130.6 (-CH=CH- *trans,trans*), 130.1 (-CH=CH- *cis,trans* + *trans,trans*), 129.5 (-CH=CH- *cis,cis*), 34.8 (-CH_2-, *trans,trans*), 32.9 (-CH_2-, *trans,trans*), 31.7 (-CH_2-, *cis,trans*), 29.5 (-CH_2-, *cis,cis*), 27.6 (-CH_2-, *cis,cis*), 23.0 (CH_2-CH_3, *cis,cis*), 22.9 (CH_2-CH_3, *cis,trans* + *trans,trans*), 14.3 (-CH_3, *cis,trans*), 13.9 (-CH_3, *cis,cis*), 13.8 (-CH_3, *trans,trans*). GC-MS (EI): m/z M^+ calculated 166.17, found 166.00.

Reaction of 4,8-dodecadiene with 1. In a glove box, 3.0 mg (0.018 mmol) of 4,8-dodecadiene (*cis/trans*) were dissolved in 0.4 ml CD_2Cl_2 and transferred into a vial. Then, 3.2 mg (8.2 μmol) of **1** dissolved in 0.2 ml CD_2Cl_2 was added in one portion. The mixture was stirred for 5 min and allowed to stand at room temperature for 18 hours.

Chloro(high *cis*-4,8-dodecadiene)rhodium(I) dimer ^{1}H-NMR (400 MHz, CD_2Cl_2, ppm): 5.42-5.35 (m, -CH=CH-, unreacted 4,8-dodecadiene), 4.70-4.15 (bm, -CH=CH-Rh), 2.85-2.75 (m), 2.57-2.50 (m), 2.45-2.20 (m), 2.09-1.22 (m), 1.10-0.85 (m).

Chloro(high *trans*-4,8-dodecadiene)rhodium(I) dimer: ^{1}H-NMR (400 MHz, CD_2Cl_2, ppm): 5.45-5.35 (m, -CH=CH-, unreacted 4,8-dodecadiene), 4.65-4.15 (bm, -CH=CH-Rh), 2.91-2.79 (m), 2.58-2.48 (m), 2.45-2.20 (m), 2.07-1.26 (m), 1.08-0.88 (m).

Kinetic study of rhodium(I) complexation to 4,8-dodecadienes. The rate of formation of chloro(4,8-dodecadiene)rhodium(I) dimer was determined by stopped-flow kinetics. For this purpose, 5 mL solution of each 4,8-dodecadiene (1.85 mM in THF) and **1** (0.60 mM in THF) were introduced into the stopped flow instrument separately in 5 mL syringes. The formation of chloro(4,8-dodecadiene)rhodium(I) dimer was monitored at 360 nm. The plot of absorbance versus time allowed us to calculate the rate constants.

Ligand exchange of chloro(4,8-dodecadiene)rhodium(I) dimer with PCy_3: In a glove box, chloro(4,8-dodecadiene)rhodium(I) dimer was prepared by mixing 6 ml 4,8-dodecadiene (1.3 mM in THF) and 1 ml of **1** (2.6 mM in THF). The solution was stirred at room temperature for 16 h. Then, 5 mL solution of each chloro(4,8-dodecadiene)rhodium(I) dimer (0.37 mM in THF) and PCy_3 (0.74 mM in THF) were introduced into the stopped flow instrument separately in 5 mL syringes. The reaction was monitored at 360 nm.

Reaction of 1 with 95% *cis*-PBD in bulk. 3 ml of 0.27 mM **1** in THF were transferred to a cuvette. Then, 8 mg of 95% *cis*-PBD were added and UV-Vis spectra were recorded.

DSC analysis of 95% *cis*-PBD with varying rhodium(I) percentage. 30.5 mg (0.1 μmol) of 95% *cis*-PBD was weighed and dissolved 2 ml of THF in two separate vials. A solution of 2 ml of **1** in THF was added to each vial in one portion (5%, 3.5 mM and 10%, 7.0 mM) and allowed to stand at room temperature. After 16 hours, the samples were evaporated *in vacuo* and DSC measurements were done.

NMR spectral data

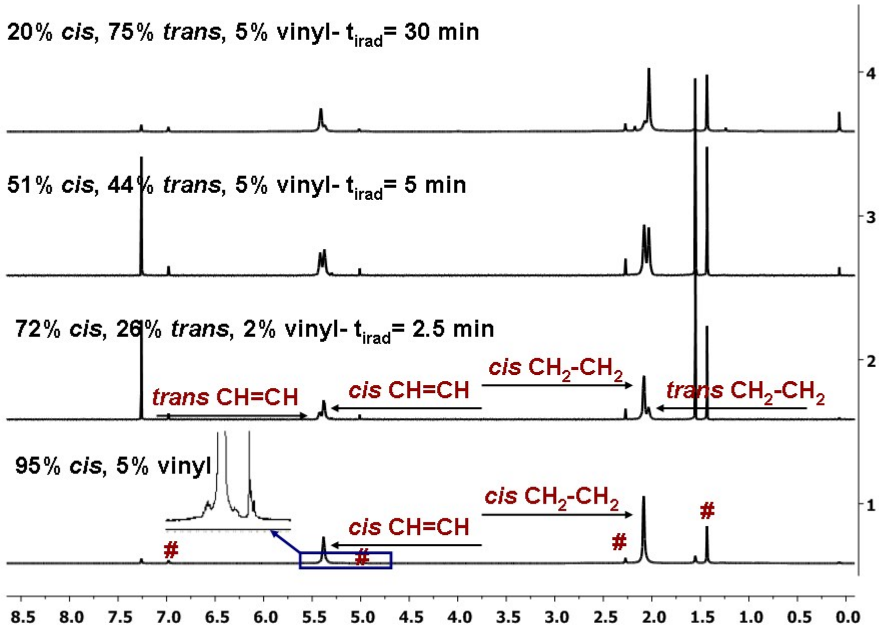

Figure S1: ^{1}H-NMR spectra of irradiated PBDs showing *cis-trans* isomerization of 95% *cis*-PBD. # = BHT

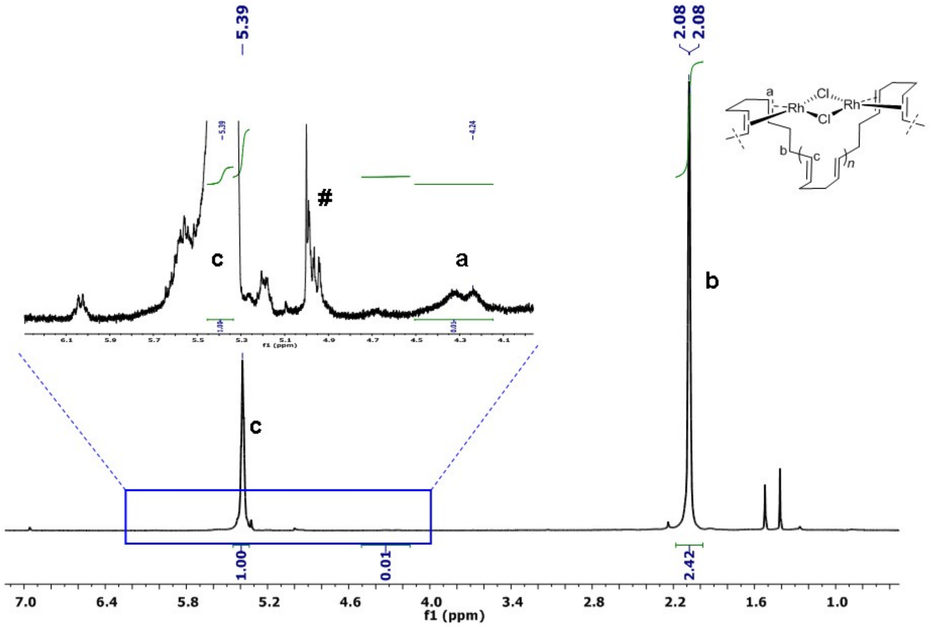

Figure S2: ^{1}H-NMR of **2** (2% Rh(I)) in CD_2Cl_2 and the expanded view of the olefinic region (Insets, # = BHT)

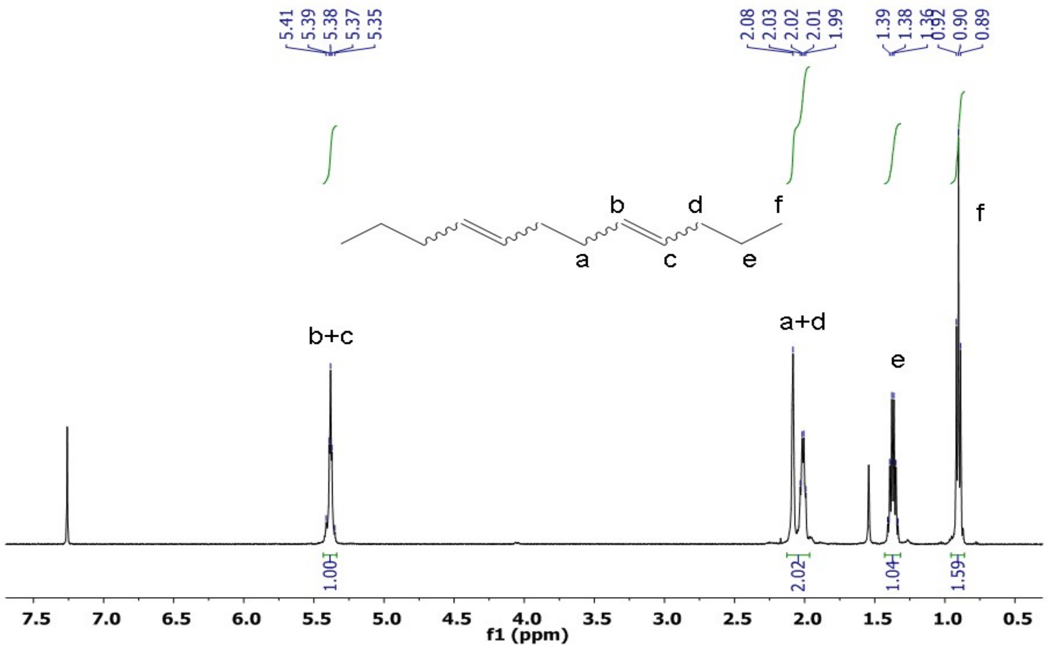

Figure S3: ^{1}H-NMR of *cis,cis*-4,8-dodecadiene in $CDCl_3$

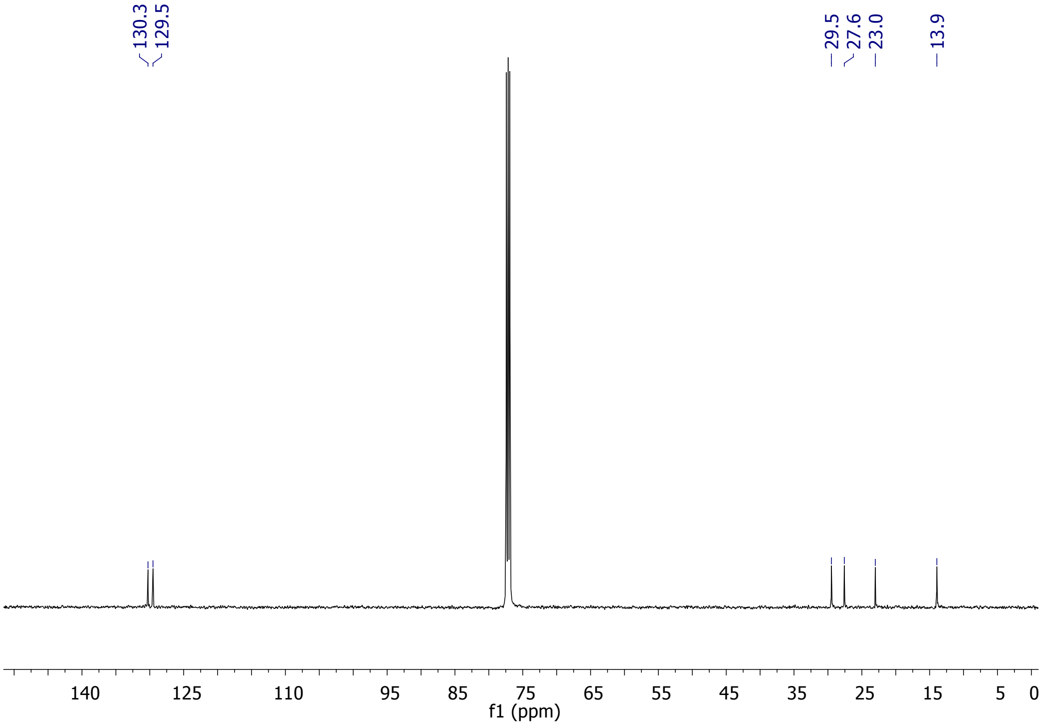

Figure S4: ^{13}C-NMR of *cis,cis*-4,8-dodecadiene in $CDCl_3$

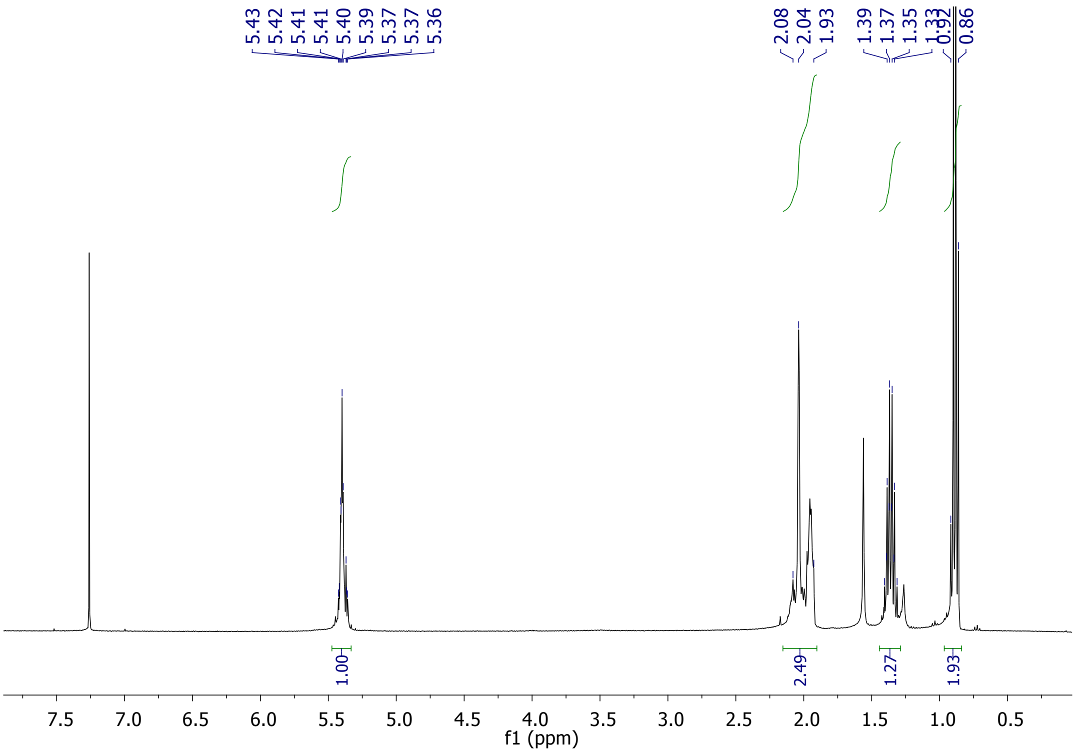

Figure S5: ^{1}H-NMR of 4,8-dodecadiene (75% *trans*) in $CDCl_3$

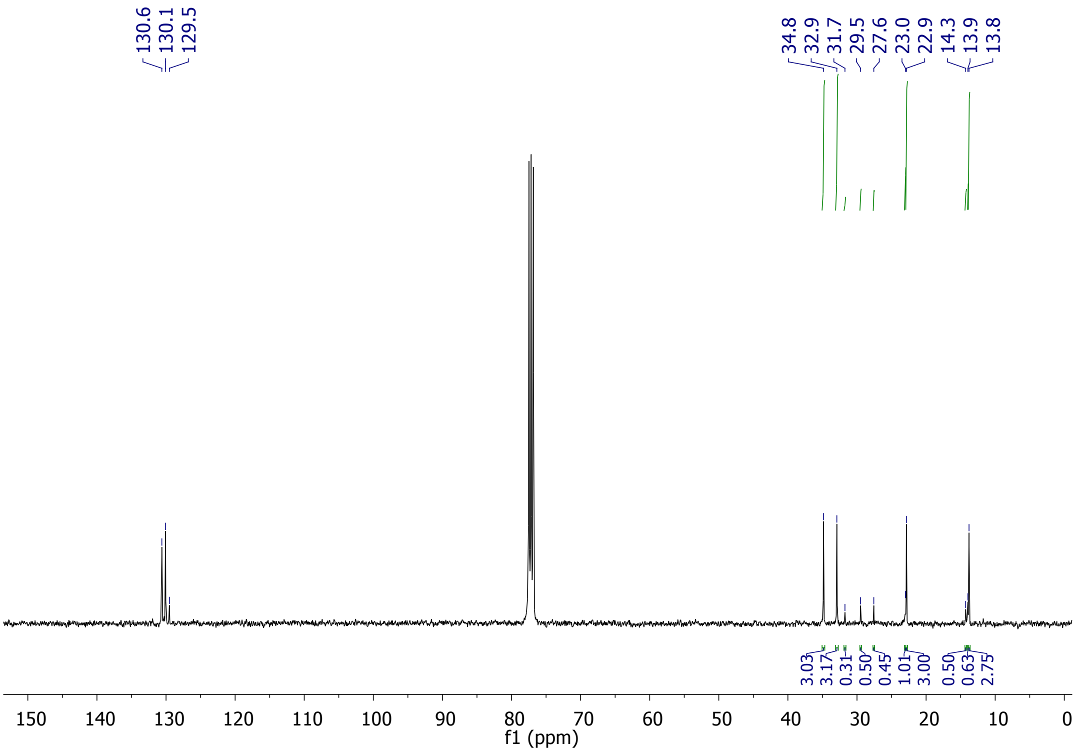

Figure S6: ^{13}C-NMR of 4,8-dodecadiene (75% *trans*) in $CDCl_3$

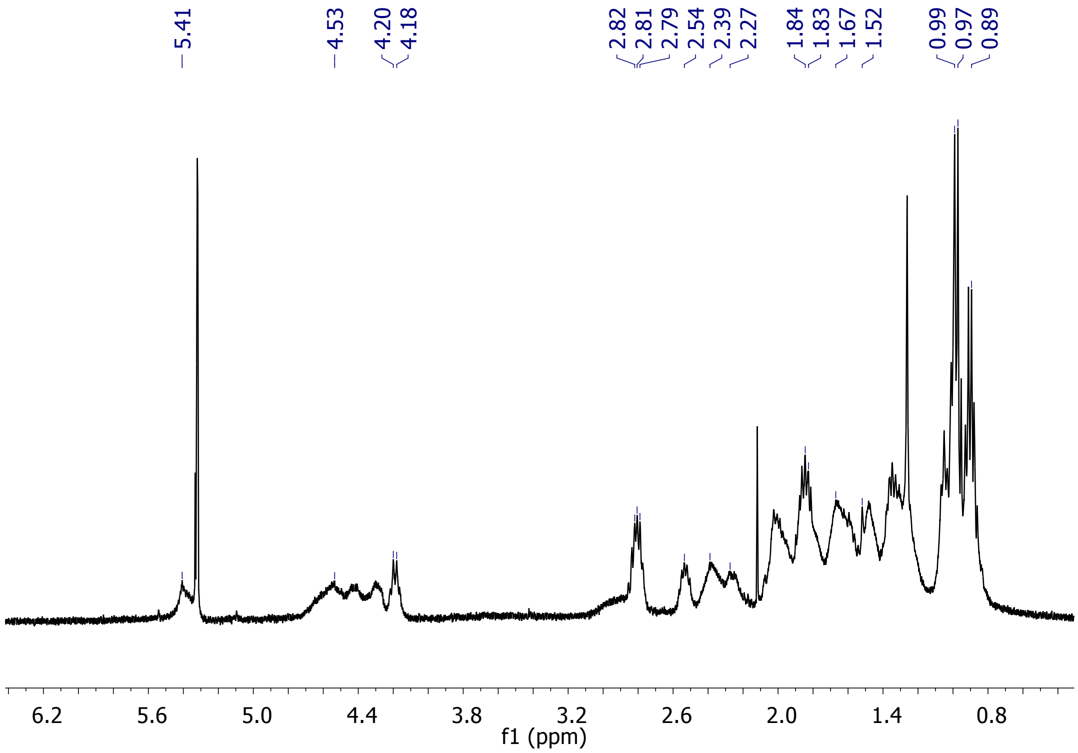

Figure S7: ^{1}H-NMR shifts in CD_2Cl_2 for the reaction of **1** with 4,8-dodecadiene (high *cis* content)

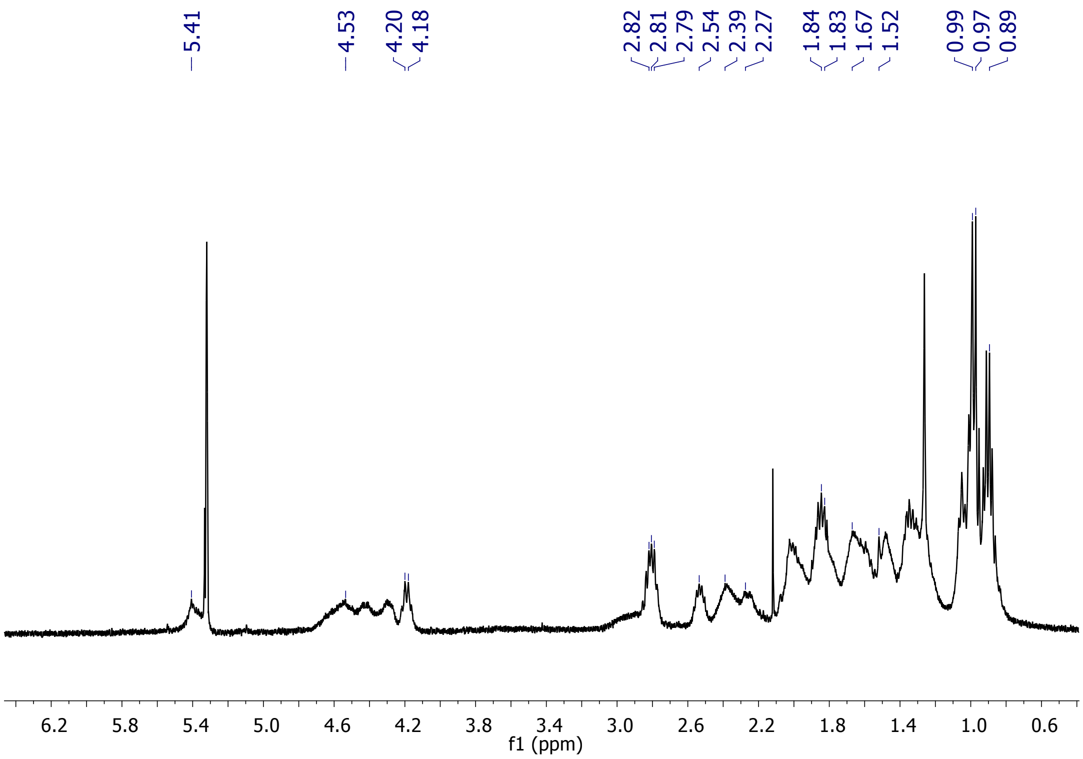

Figure S8: ^{1}H-NMR shifts in CD_2Cl_2 for the reaction of **1** with 4,8-dodecadiene (~75% *trans*)

SEC and DLS data

Table S1: SEC measurements of PBD with varying *cis* content.

#	***cis*** **(%)**[a]	M_w **[× 10^5 gmol^{-1}]**[b]	**PDI**	**Intrinsic viscosity (mLg^{-1})**[b]	R_h **(nm)**[b]
1	95	3.14	2.47	168.1	18.7
2	72	3.66	2.11	192.5	20.7
3	51	3.49	1.98	198.6	20.7
4	20	4.04	1.44	213.8	22.6

[a]Determined by ^{1}H-NMR,[b]Determined by triple-detector SEC in THF. Values calculated assuming dn/dc=0.129 mLg^{-1}.[4]

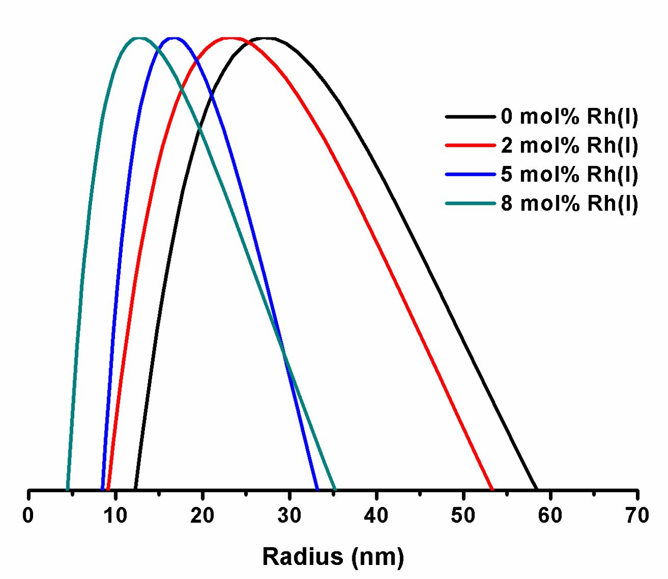

Figure S9: Overlay of DLS traces obtained for 95%-*cis* Rh(I)-ONPs at varying concentrations of Rh(I).

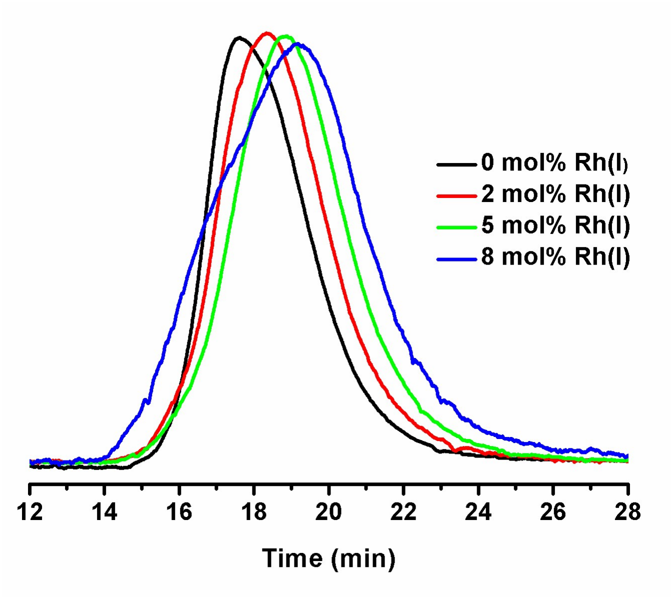

Figure S10: Overlay of the SEC plots obtained for 72%-*cis* Rh(I)-ONPs at varying concentration of Rh(I).

Table S2: SEC analysis obtained for 72%-*cis* Rh(I)-ONPs at varying concentration of Rh(I)[a]

#	**Rh(I) [mol%]**[b]	M_w **[× 10^5 gmol^{-1}]**[b]	**PDI**	**Intrinsic viscosity (mLg^{-1})**[b]	R_h **(nm)**[b]
1	0	3.60	2.12	192.5	20.7
2	2	2.91	1.81	161.0	18.5
3	5	2.89	1.61	116.0	16.6
4	8	2.28	1.73	73.6	13.2

[a]Conditions: solvent = THF, t = 16 h, T = 35 °C, Rh(I) = $[RhCl(C_2H_4)_2]_2$, [b]Relative to 1,5-hexadiene units. [c]Determined by triple-detector SEC in THF.

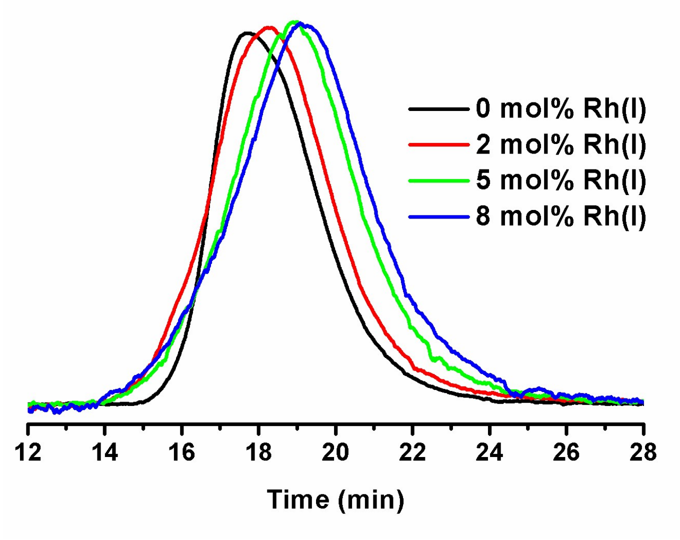

Figure S11: Overlay of the SEC plots obtained for 51%-*cis* Rh(I)-ONPs at varying concentration of Rh(I)

Table S3: SEC analysis obtained for 51%-*cis* Rh(I)-ONPs at varying concentration of Rh(I)[a]

#	**Rh(I) [mol%]**[b]	$\boldsymbol{M_w}$ **[× 10^5 gmol^{-1}]**[b]	**PDI**	**Intrinsic viscosity (mLg^{-1})**[b]	$\boldsymbol{R_h}$ **(nm)**[b]
1	0	3.49	1.98	198.6	20.7
2	2	3.09	1.72	151.6	18.4
3	5	2.75	1.49	108.7	16.1
4	8	3.96	1.43	106.7	17.9

[a]Conditions: solvent = THF, t = 16 h, T = 35 °C, Rh(I) = $[RhCl(C_2H_4)_2]_2$, [b]Relative to 1,5-hexadiene units. [c]Determined by triple-detector SEC in THF.

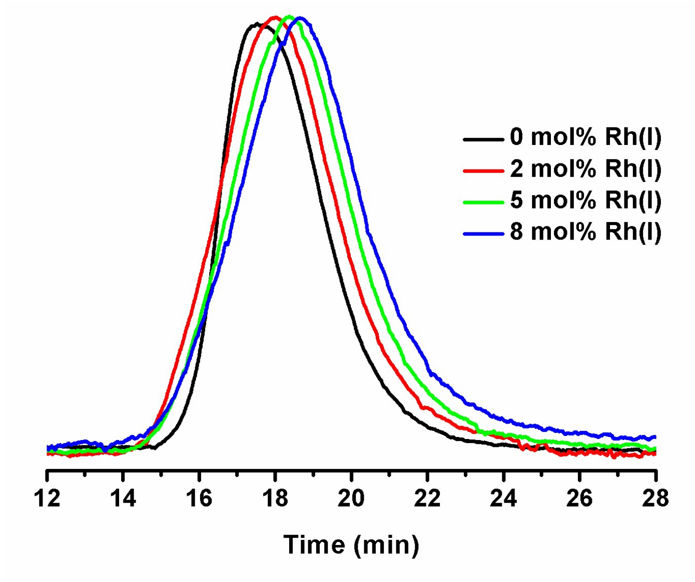

Figure S12: Overlay of the SEC plots obtained for 20%-*cis* Rh(I)-ONPs at varying concentration of Rh(I).

Table S4: SEC analysis obtained for 20%-*cis* Rh(I)-ONPs at varying concentration of Rh(I)[a]

#	Rh(I) [mol%][b]	M_w [× 10^5 $gmol^{-1}$][b]	PDI	Intrinsic viscosity (mLg^{-1})[b]	R_h (nm)[b]
1	0	4.04	1.45	213.8	22.6
2	2	3.16	1.42	161.5	19.3
3	5	2.66	1.47	129.4	17.0
4	8	2.60	1.68	113.8	15.9

[a]Conditions: solvent = THF, t = 16 h, T = 35 °C, Rh(I) = $[RhCl(C_2H_4)_2]_2$, [b]Relative to 1,5-hexadiene units. [c]Determined by triple-detector SEC in THF.

UV and stopped flow data

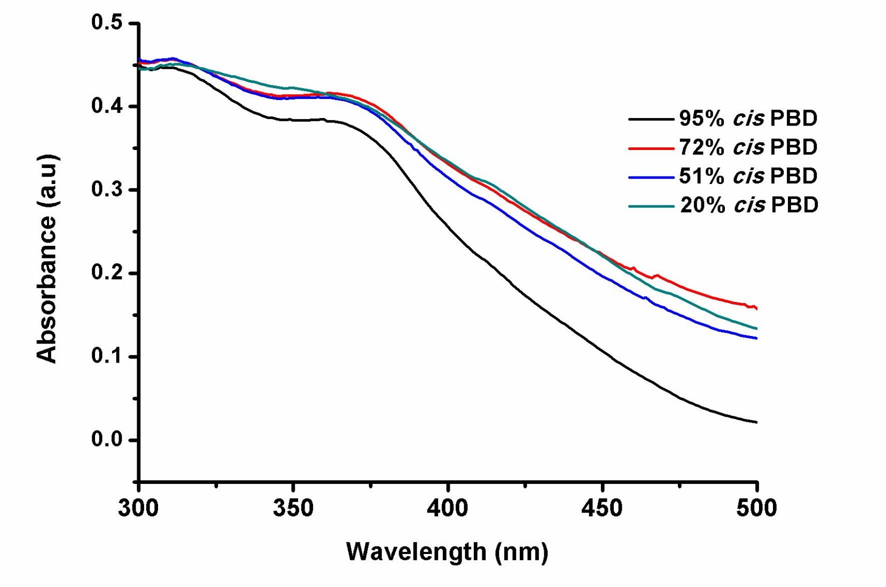

Figure S13: UV-Vis spectra of Rh(I)-ONPs made from PBD with varying *cis* content

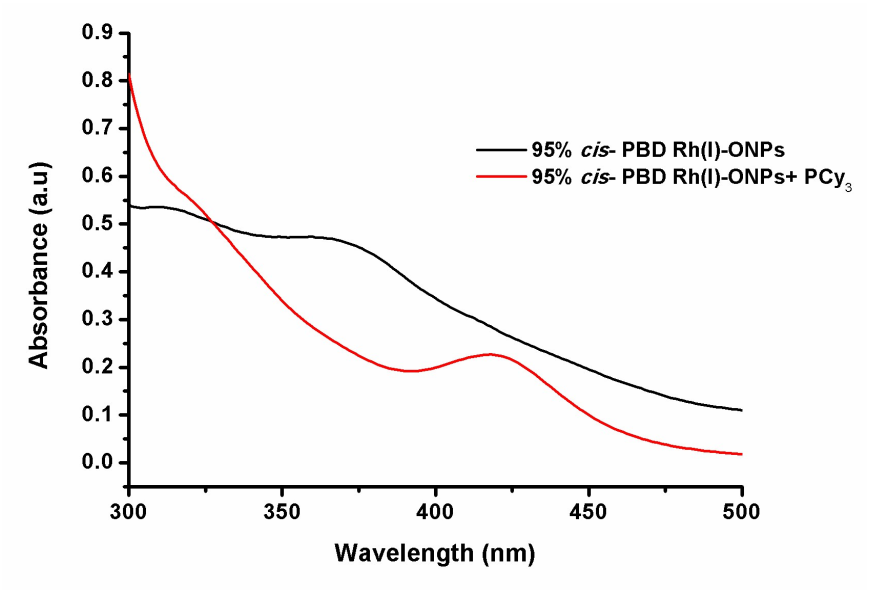

Figure S14: UV spectra of 95%-*cis* Rh(I)-ONP(10 mol%) before and after addition of PCy_3

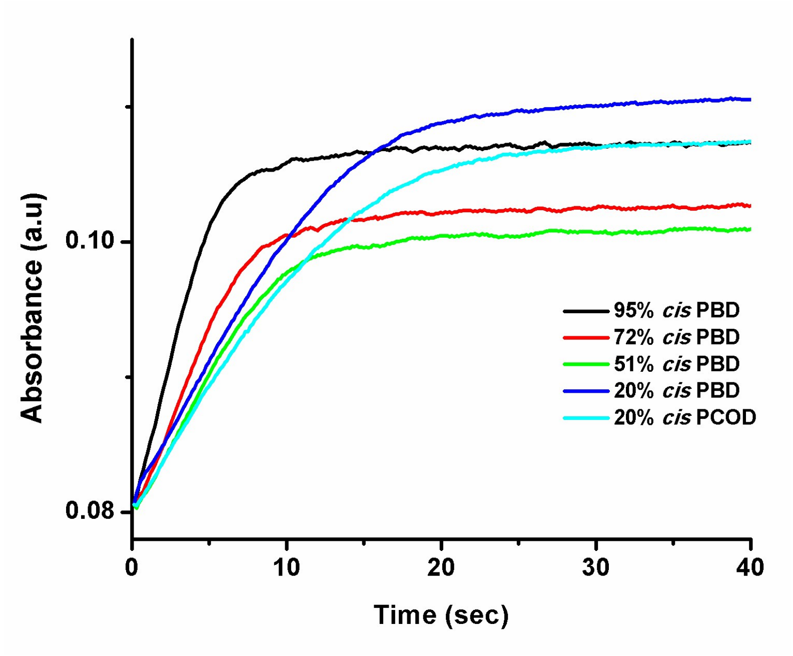

Figure S15: Plot of absorbance versus time obtained for formation of Rh(I)-ONP made of PBD and PCOD with varying *cis* content using stopped flow kinetics

Table S5: Rate constants for the formation of Rh(I)-ONPs

Polymer	95% *cis*-PBD	72% *cis*-PBD	51% *cis*-PBD	20% *cis*-PBD	20% *cis*-PCOD
k (s^{-1})	0.3195 ±0.0025	0.2409 ±0.0025	0.1937 ±0.0016	0.1178 ±0.0010	0.1102 ±0.0008

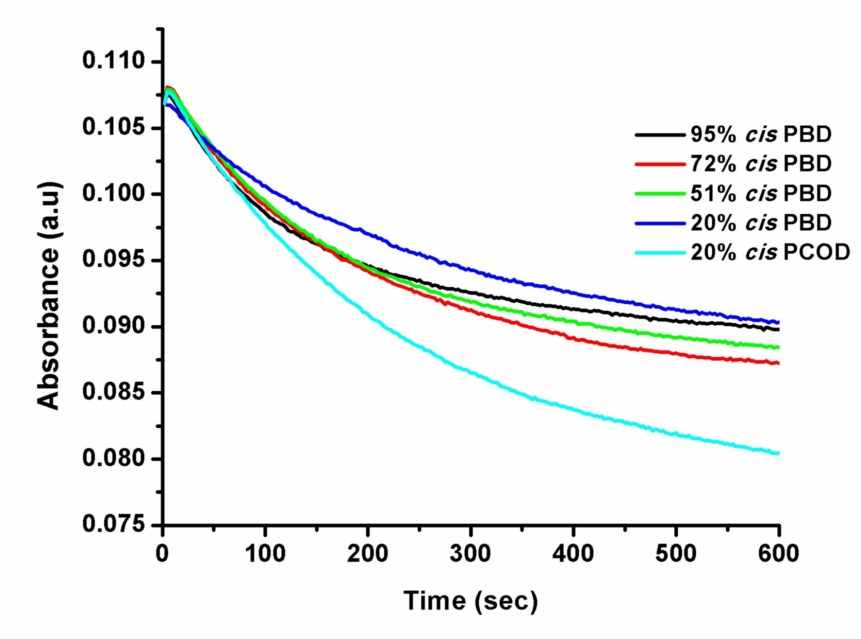

Figure S16: Plot of absorbance versus time obtained the reaction of different *cis* content Rh(I)-ONP with PCy_3 using stopped flow kinetics

Table S6: Rate constants for the reaction of Rh(I)-ONPs with PCy_3

Polymer	95% *cis*-PBD	72% *cis*-PBD	51% *cis*-PBD	20% *cis*-PBD	20% *cis*-PCOD
k (s^{-1})	0.00593 ±0.00005	0.00498 ±0.00002	0.00512 ±0.00003	0.00366 ±0.00002	0.00417 ±0.00001

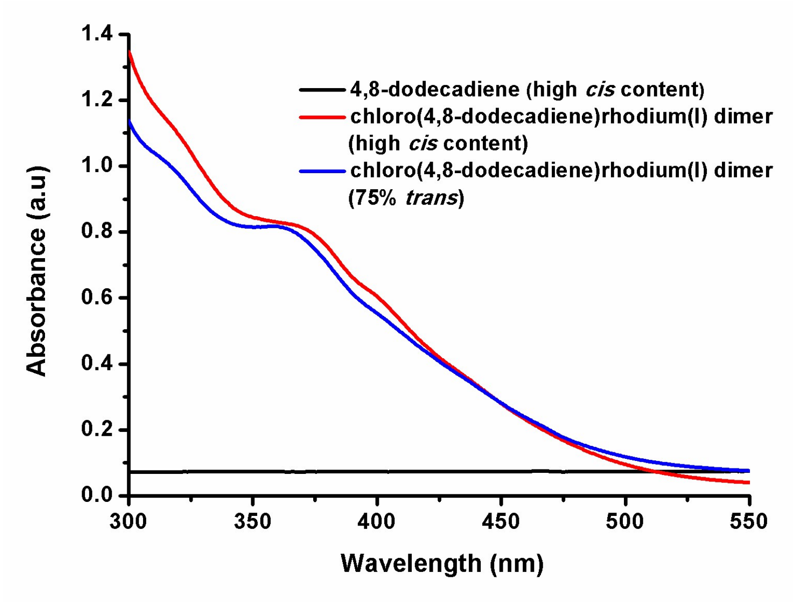

Figure S17: UV-Vis spectra of chloro(4,8-dodecadiene)rhodium(I) dimer in THF

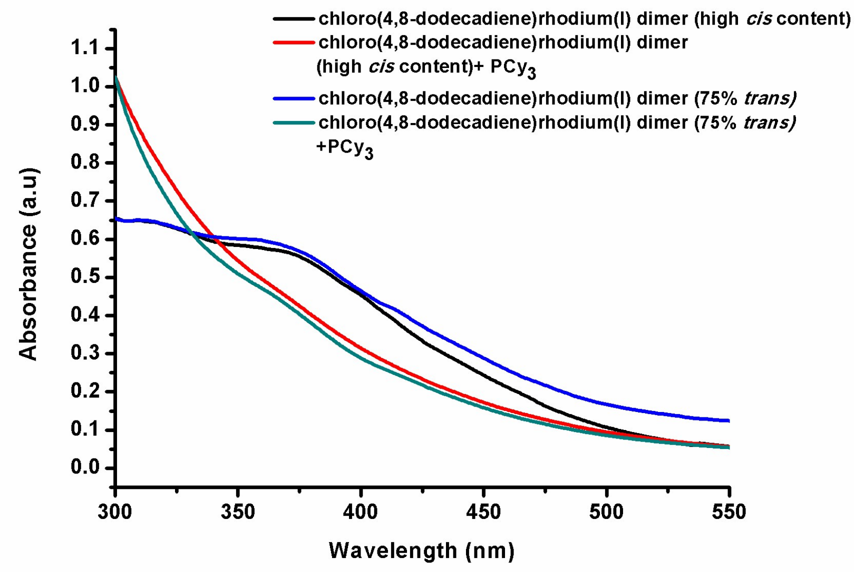

Figure S18: UV-Vis spectra of chloro(4,8-dodecadiene)rhodium(I) dimer prior and following the addition of PCy_3 in THF

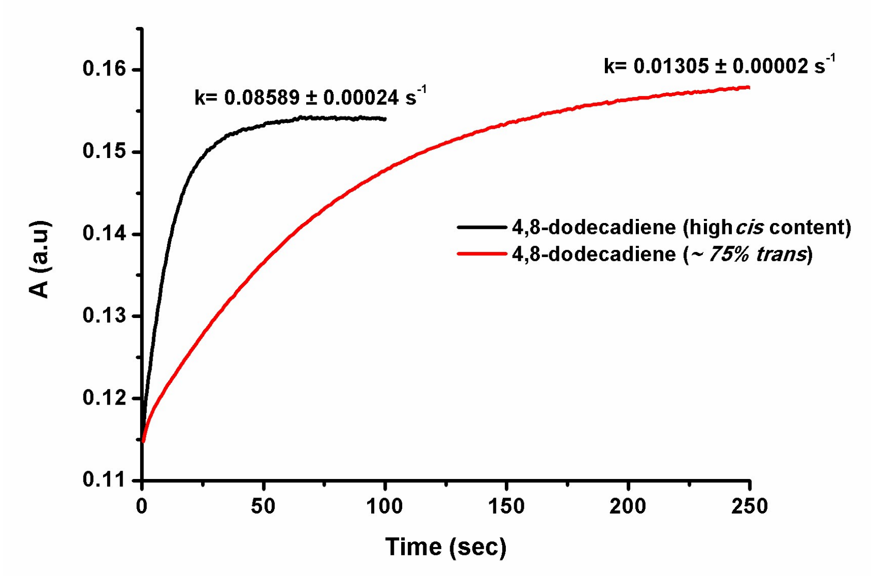

Figure S19: Plot of absorbance versus time obtained for the formation of chloro(4,8-dodecadiene)rhodium(I) dimer using stopped flow kinetics.

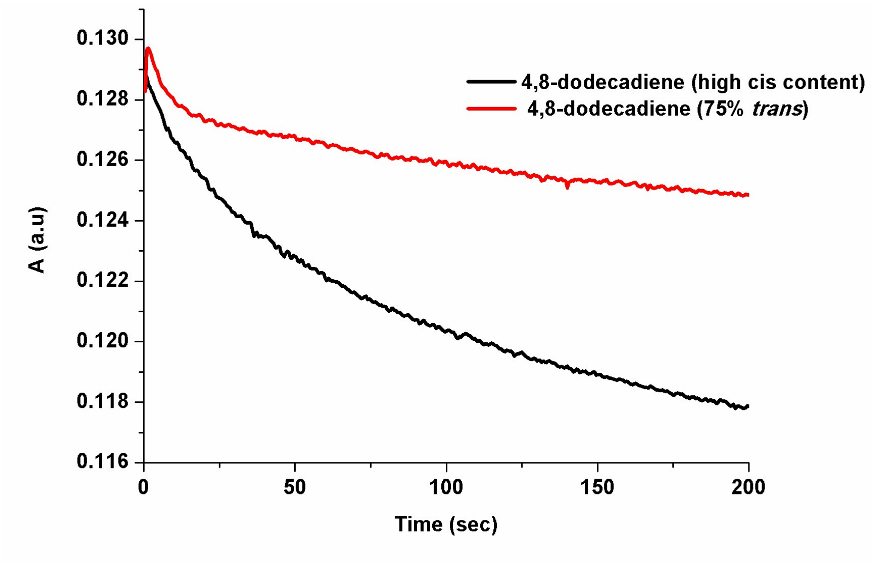

Figure S20: Plot of absorbance versus time obtained for the reaction of chloro(4,8-dodecadiene)rhodium(I) dimer with PCy_3 using stopped flow kinetics.

Table S7: Rate constants for the reaction of chloro(4,8-dodecadiene)rhodium(I) dimer with PCy_3

4,8-dodecadiene type	High *cis* content	75% *trans*
k (s^{-1})	0.0128 ± 0.0001	Single exponent: 0.0130 ± 0.0005<br>Double exponent:<br>k_1= 0.2174 ± 0.0063<br>k_2: 0.0065 ± 0.0002

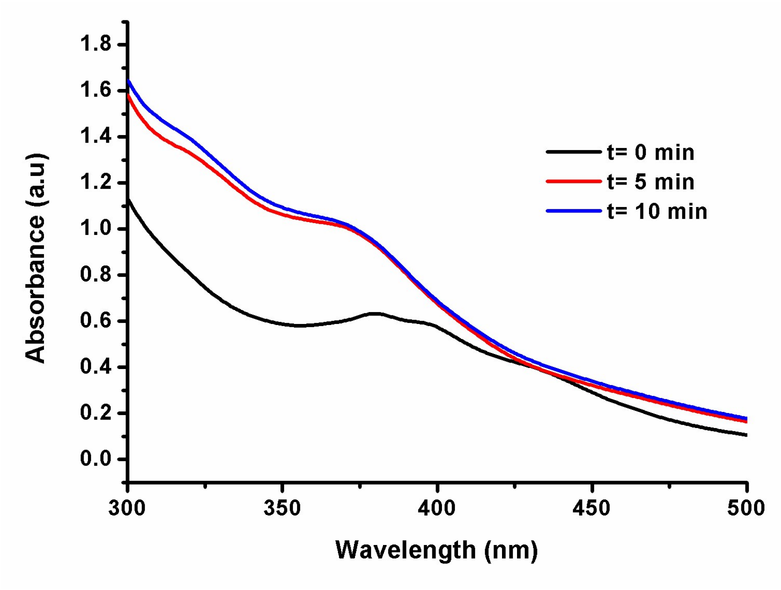

Figure S21: UV-Vis spectra of **1** with solid 95% *cis*-PBD in THF

GC-MS chromatograms

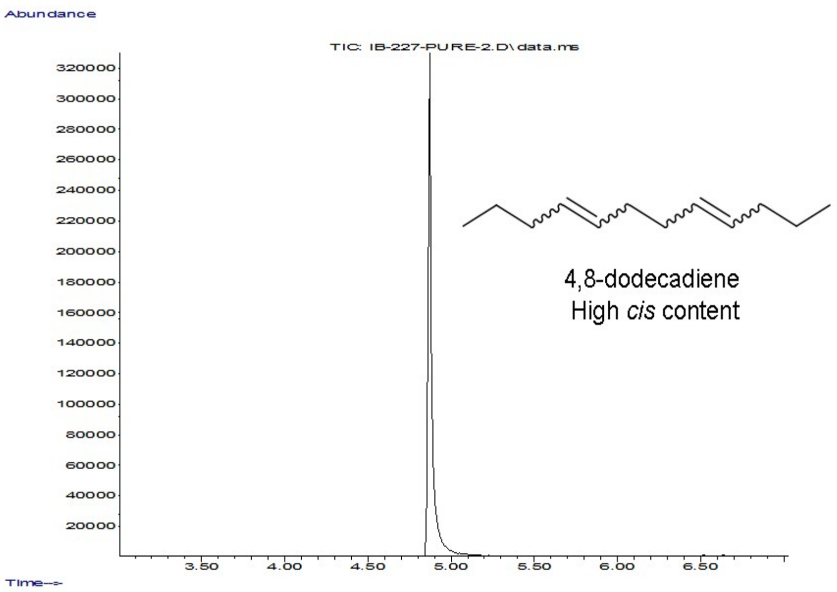

Figure S22: GC-MS chromatogram of high *cis* content 4,8-dodecadiene

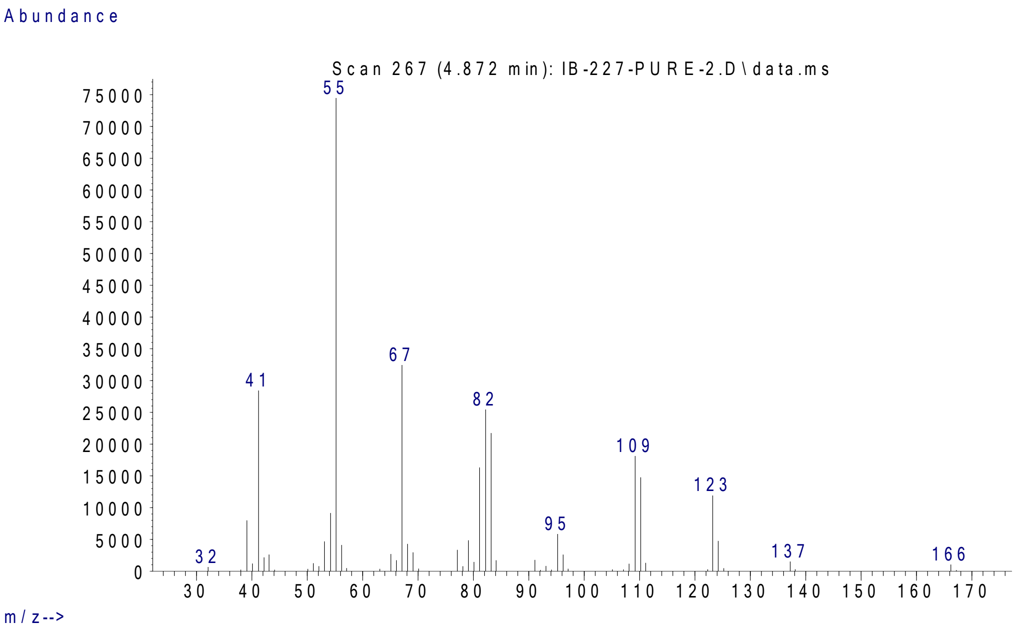

Figure S23: MS spectrum of high *cis* content 4,8-dodecadiene.

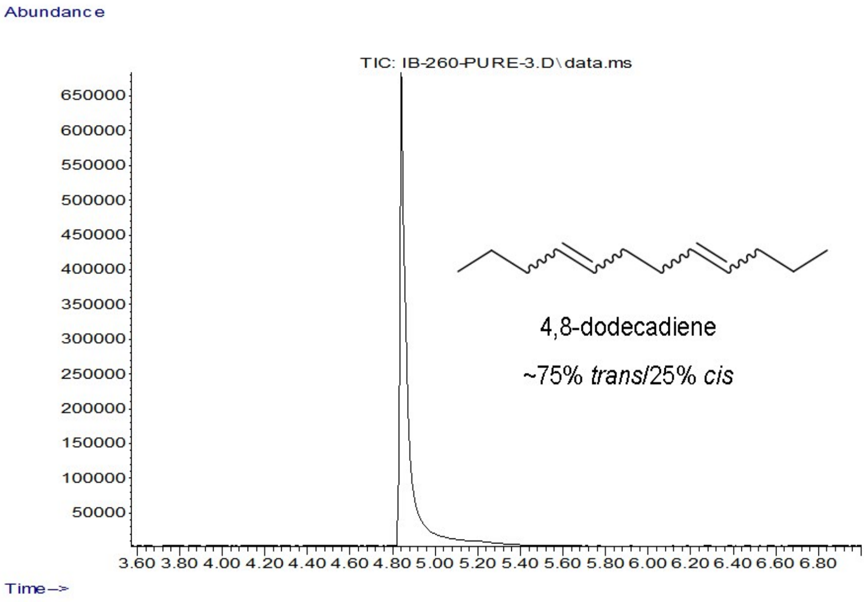

Figure S24: GC-MS chromatogram of ~ 75% *trans* 4,8-dodecadiene.

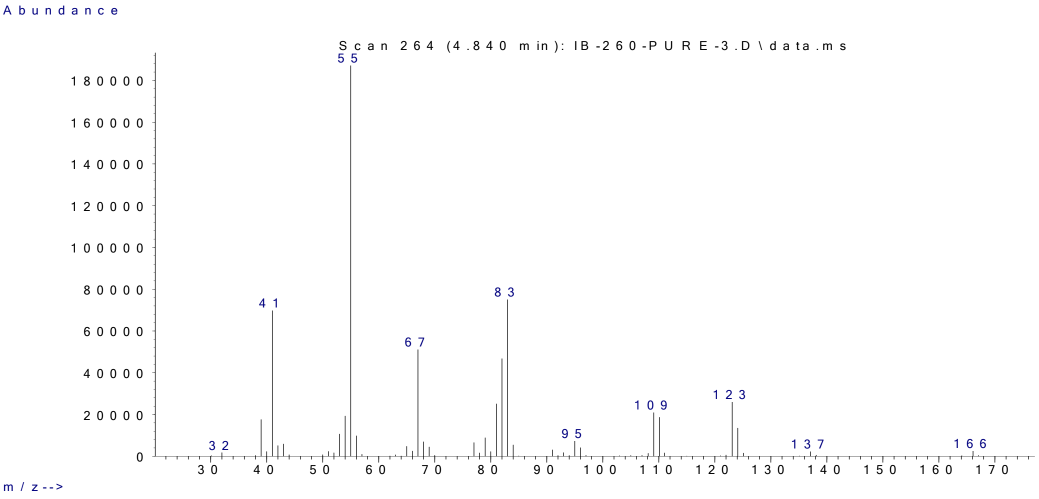

Figure S25: MS spectrum of ~ 75% *trans* 4,8-dodecadiene.

DSC and TGA data

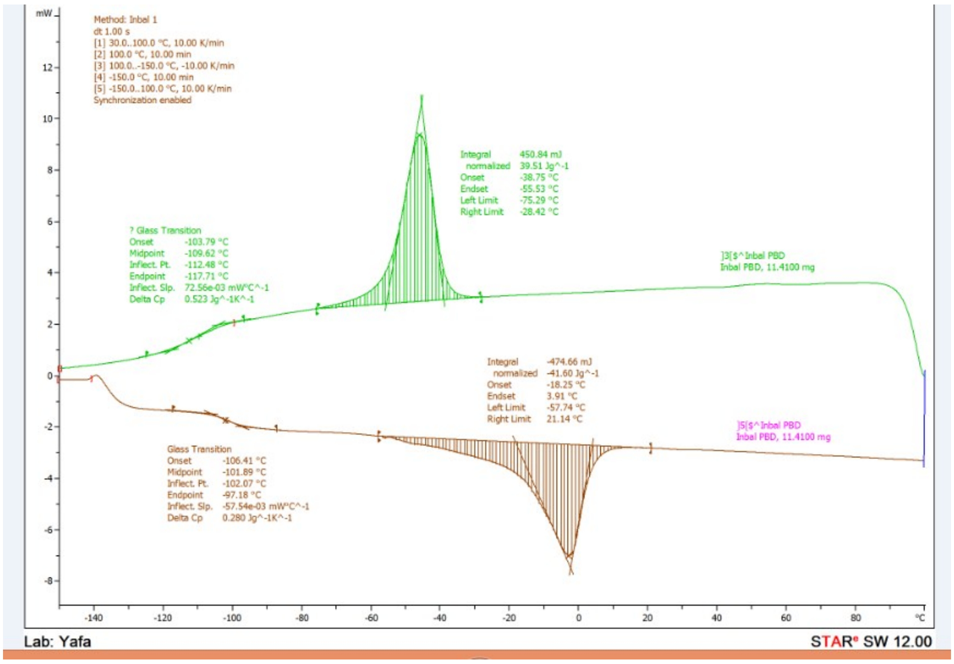

Figure S26: DSC plot of 95% *cis*-PBD

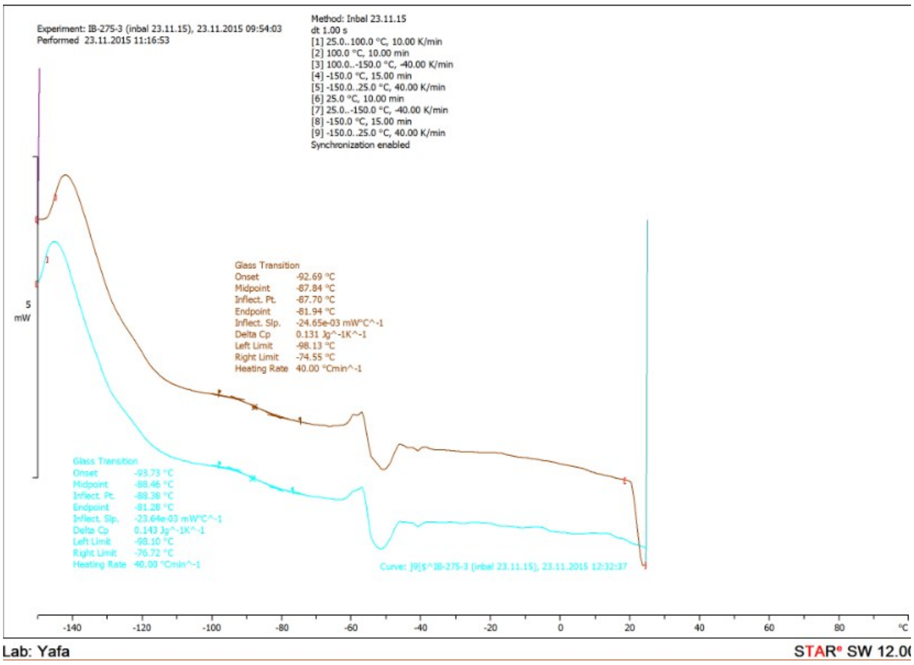

Figure S27: DSC plot of 95% *cis*-PBD with 5% Rh(I)

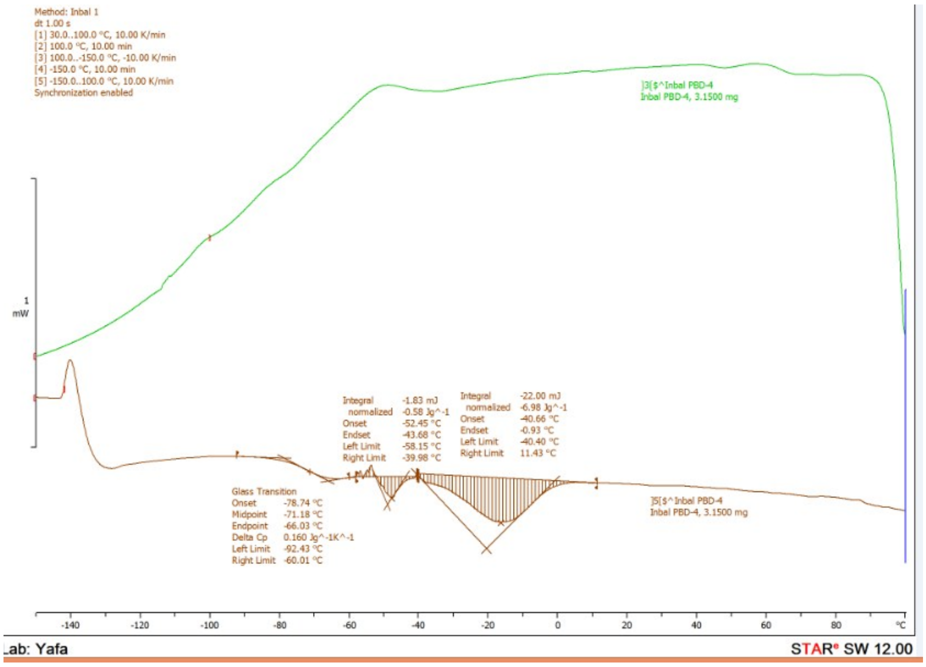

Figure S28: DSC plot of 95% *cis*-PBD with 10% Rh(I)

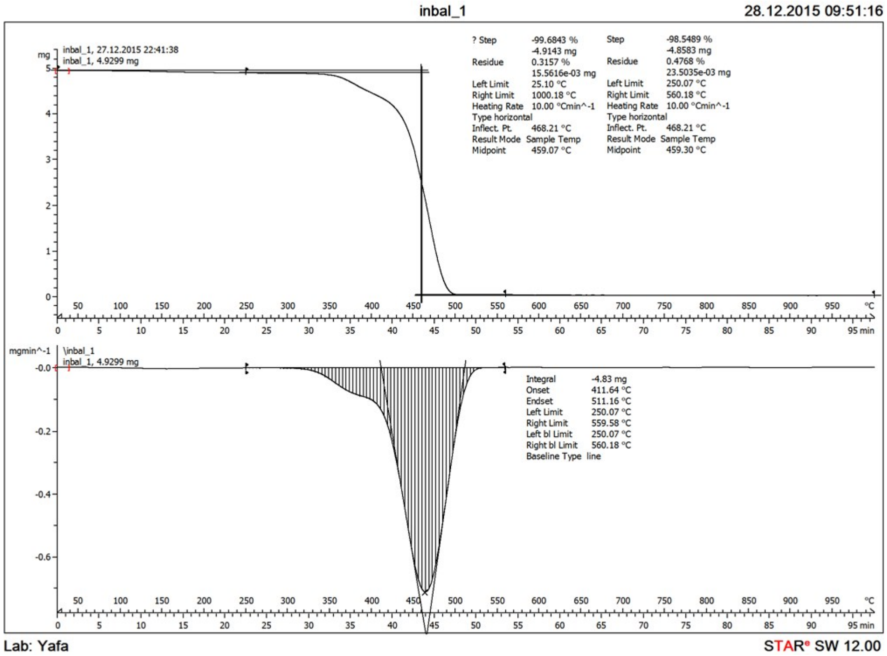

Figure S29: TGA plot of 95% *cis*-PBD

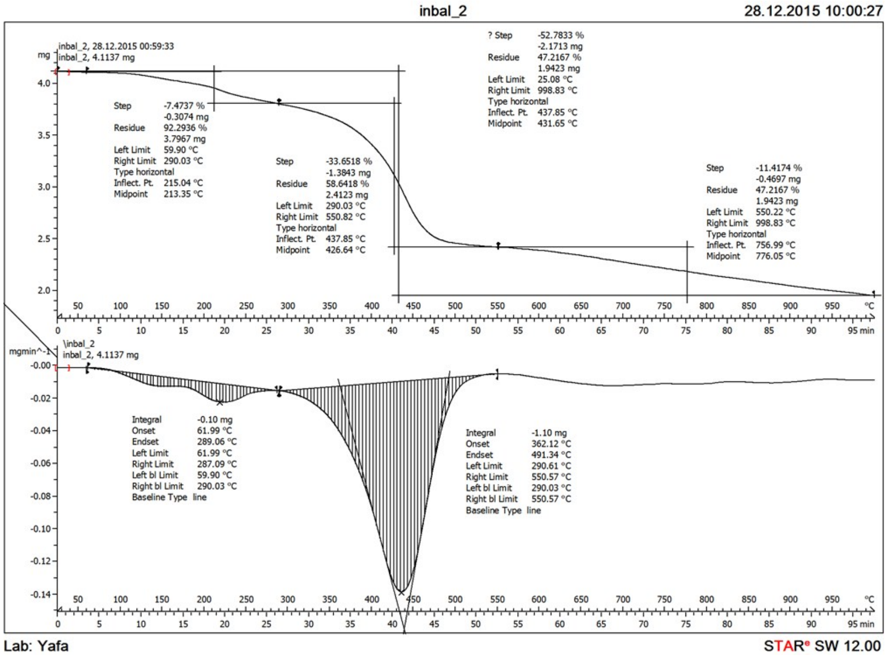

Figure S30: TGA plot of 95% *cis*-PBD with 5% Rh(I)

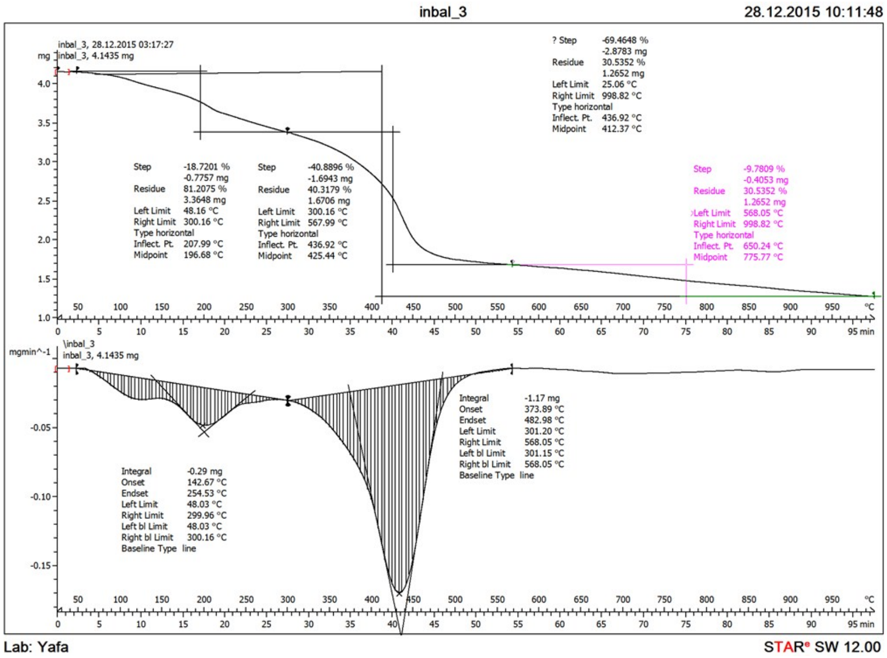

Figure S31: TGA plot of 95% *cis*-PBD with 10% Rh(I)

TGA analysis has shown that 95% *cis*-PBD has a single degradation step with a midpoint of 459.1 °C. As more rhodium was added to the polymer, the midpoint of this step was shifted to lower temperatures (431.7 °C and 412.4 °C for 5% and 10%, respectively). In addition, the added rhodium led to another degradation step at even lower temperatures - in the 5% Rh(I) sample, 7.5% of the sample was degraded at 213.4 °C and in the 10% Rh(I) sample 18.7% of the sample was degraded at 196.7 °C

Computational calculations of rhodium binding and release

Theoretical Method:

The B97D3BJ method of Grimme was selected for geometry optimizations[5,6], since it provides fast but also reliable geometries for transition metal complexes. It includes the most advanced dispersion correction of Grimme's group, and being a pure GGA, density fitting correction can be used to significantly accelerate the computations. The Def2-SVP basis set is not always accurate enough for energies, but it is adequate and inexpensive for geometries, and therefore it was used for all the optimizations. Frequencies (and subsequent Gibbs energy corrections) where computed at this same level, using a pressure of 24.5 atm to simulate standard 1 M concentrations in a solvent.

Single point energies were computed with the M06 functional of Truhlar and co-workers[7,8]. This functional has a balanced exact exchange to compute reactions involving transition metals, and can accurately include medium-range dispersion interactions. The employed Def2-TZVPP basis set is an accurate triple-zeta adequate for DFT energies (where basis set convergence is much faster than with post-HF ab initio methods). It must be pointed out that preliminary tests with ωB97XD, another accurate hybrid functional for this type of reactions, provided very similar results (and virtually the same selectivity).

Solvent (THF) computations where included with the SMD method of Truhlar et al. This method provides good solvation Gibbs energies, and thus it is more adequate for Gibbs energies (the values expressed in the article) than for internal energies. Nevertheless, as can be seen from table S8, the relative kinetics of the *cis* and *trans* butene ligand substitution are almost unaffected by the solvent, as the solvation energy is practically identical for both reactants.

In the paper it is argued that the association energy for the double *cis* octadiene is less exothermic than for the double *trans* conformer. It must be noted that there is another *cis*-diene complex conformation ("conf. II" in table S8, see its xyz geometry below) with stronger association energy than the one depicted in Fig. 6 of the main text. This conformer is still less stable than the *trans* case, so the postulated explanation of the faster *cis* diene dissociation still stands when considering this alternative *cis* conformation.

Nonetheless, it is much harder to obtain the conf. II through a stepwise substitution of the ethene groups, and therefore we did not consider it further.

As explained in the main text, breaking the verticality of the olefin ligand diminishes the back-bonding ability of the metal to the ligand, generating a more labile bond. To quantitatively analyse this effect, we studied the energy required to twist the dihedral angle of the double bond in a model *trans*-$Rh(PH_3)_2Cl(H_2C{=}CH_2)$ complex with respect to the complex plane. By twisting the ligand by 10° and 20°, the energy of the complex rises by 5.6 and 11.0 kJ/mol, respectively. This shows that the stronger steric impediment felt by the *trans*-butene indeed generates a significant effect on the stability and ligand substitution kinetics of the Rh complex.

Table S8. Absolute and relative energies of the computed systems (see geometries below). An "Inverted" conformation corresponds to the systems with an inversion of the angle between the coordination planes of the two metal atoms. For the *trans*-butene system the olefin can be coordinated in two ways, as can be seen in Fig. 4 of the main text; "conf II" has a slightly higher TS_{cd}, and therefore corresponds to the less crossed pathway (hence not discussed in the article).

		Absolute Energies (Ha)				Relative Energies (kJ/mol) M06/def2-tzvpp// b97d3/def2-svp			
**Structure**	**Conformation**	**B97D3BJ Def2-SVP**	**Gibbs Energy Correction (B97D3BJ)**	**M06 Def2-TZVPP (gas phase)**	**M06 Def2-TZVPP (THF)**	**E gas**	**E THF**	**G gas**	**G THF**
1a		-1455.97258	0.17519	-1456.00448	-1456.01766				
2-butene	*cis*	-157.01152	0.08104	-157.14665	-157.15069				
2-butene	*trans*	-157.01292	0.08129	-157.14818	-157.15219				
ethene		-78.48054	0.03182	-78.54802	-78.54920				
Octadiene	*cis*	-312.82401	0.15892		-313.10455				
Octadiene	*trans*	-312.82645	0.16004		-313.10748				
1b	*cis*	-1612.99770	0.27122	-1613.15978	-1613.17557	-22.7	-19.0	16.7	20.4
TSbc	*cis*	-1612.99212	0.27475	-1613.15025	-1613.16384	2.3	11.8	50.9	60.5
1c	*cis*	-1612.99393	0.27363	-1613.15271	-1613.16630	-4.2	5.4	41.5	51.1
TScd	*cis*	-1612.98732	0.27446	-1613.14516	-1613.15877	15.7	25.1	63.5	73.0

1d	*cis*	-1612.99327	0.26950	-1613.15463	-1613.16909	-9.2	-2.0	25.7	32.9
RhEtBu + Et	*cis*	-1612.98348	0.25661	-1613.14759	-1613.16372	9.3	12.2	10.3	13.2
1e	*cis*	-1534.50295	0.22479	-1534.59957	-1534.61452	9.3	12.2	10.3	13.2
TScd	*cis* - Inverted	-1612.98652	0.27420	-1613.14287	-1613.15767	21.7	28.0	68.9	75.2
TScd	*trans*	-1612.98715	0.27514	-1613.14495	-1613.15951	20.3	27.1	69.2	76.1
1e	*trans*	-1534.50222	0.22410	-1534.59954	-1534.61494	13.4	15.0	11.9	13.5
TScd	*trans*-Conf II	-1612.98599	0.27459	-1613.14427	-1613.15766	22.0	32.0	69.6	79.5
1e	*trans*-Conf II	-1534.50253	0.22550	-1534.60034	-1534.61529	11.3	14.1	13.5	16.2
TScd	*trans*-Conf II-Inverted	-1612.98549	0.27340	-1613.14224	-1613.15665	27.4	34.7	71.8	79.1
TScd	*trans*-Inverted	-1612.98533	0.27310	-1613.14235	-1613.15705	27.1	33.6	70.7	77.2
$Rh_2Cl_2Et_2$-Octadiene	*cis*	-1611.81217	0.25938		-1611.99991		66.2		33.6
$Rh_2Cl_2Et_2$-Octadiene	*cis* -Conf II	-1611.82200	0.25851		-1612.00826		44.3		9.7
$Rh_2Cl_2Et_2$-Octadiene	*trans*	-1611.83223	0.26073		-1612.01690		25.8		-2.7

XYZ Geometries computed at B97D3BJ/Def2-SVP level with density fitting approximation.

Example of route line: #p b97d3/def2svp/auto opt pressure=24.5 freq EmpiricalDispersion=GD3BJ

***cis*-2-Butene (C2v)**

C	0.000000	0.674177	0.665707
H	0.000000	1.176310	1.645730
C	0.000000	-0.674177	0.665707
H	0.000000	-1.176310	1.645730
C	0.000000	1.594166	-0.523182
H	0.000000	1.054974	-1.484975
H	-0.885825	2.259598	-0.507952
H	0.885825	2.259598	-0.507952
C	0.000000	-1.594166	-0.523182
H	0.000000	-1.054974	-1.484975
H	0.885825	-2.259598	-0.507952
H	-0.885825	-2.259598	-0.507952

***trans*-2-Butene (C2h)**

C	0.324939	0.589034	0.000000
H	1.427891	0.578494	0.000000
C	-0.324939	-0.589034	0.000000
H	-1.427891	-0.578494	0.000000
C	-0.324939	1.943057	0.000000
H	-0.022280	2.536140	0.886080
H	-0.022280	2.536140	-0.886080
H	-1.427093	1.868317	0.000000
C	0.324939	-1.943057	0.000000
H	1.427093	-1.868317	0.000000
H	0.022280	-2.536140	0.886080
H	0.022280	-2.536140	-0.886080

Ethene (D2h)

C	0.000000	0.000000	0.669749
C	0.000000	0.000000	-0.669749

1b *cis*

Rh	1.705411	-0.618444	-0.138842
C	3.026244	0.576830	-1.273153
C	3.074144	-1.651834	1.095404
C	3.100636	-0.764148	-1.714766
Cl	-0.141630	-1.361826	1.326987
H	2.414660	1.306837	-1.818383
C	2.934295	-0.319233	1.546989
H	3.837214	1.012634	-0.675294
C	1.255766	3.175198	0.193931
H	3.971048	-1.385348	-1.466796
H	2.524386	-2.468062	1.583563
H	2.545378	-1.088675	-2.605502
H	2.277993	3.432272	-0.122772
H	3.984786	-1.964299	0.567724
C	1.100219	2.710256	1.450377
H	3.729688	0.417318	1.375563
H	2.266751	-0.086723	2.387264
H	2.004691	2.633876	2.072519
C	-0.175606	2.283723	2.114855
H	-1.025065	2.239021	1.413142
H	-0.066524	1.274559	2.552176
H	-0.444280	2.968256	2.944407
Cl	-0.089533	-0.248952	-1.795744
Rh	-1.882576	-0.372690	-0.110307
C	0.187987	3.384555	-0.839630
H	0.339136	2.716578	-1.709593
H	-0.822294	3.187171	-0.447326
H	0.209552	4.423098	-1.223687
C	-3.355428	-1.264976	1.113012
C	-3.069359	0.032579	1.593505

H	0.000000	0.934602	-1.247441
H	0.000000	-0.934602	-1.247441
H	0.000000	-0.934602	1.247441
H	0.000000	0.934602	1.247441

1a (C2v)

Rh	0.000000	1.528794	-0.090266
Rh	0.000000	-1.528794	-0.090266
Cl	-1.645335	0.000000	-1.092830
Cl	1.645335	0.000000	-1.092830
C	1.488552	3.018396	0.112946
C	1.480134	2.200571	1.265479
C	-1.480134	2.200571	1.265479
C	-1.488552	3.018396	0.112946
H	-2.225927	2.850102	-0.683518
H	-1.072158	4.033447	0.150826
H	-1.065137	2.575656	2.209920
H	-2.209470	1.386876	1.372250
H	1.065137	2.575656	2.209920
H	2.209470	1.386876	1.372250
H	2.225927	2.850102	-0.683518
H	1.072158	4.033447	0.150826
H	-1.065137	-2.575656	2.209920
H	1.065137	-2.575656	2.209920
H	-1.072158	-4.033447	0.150826
H	1.072158	-4.033447	0.150826
C	1.488552	-3.018396	0.112946
C	1.480134	-2.200571	1.265479
C	-1.480134	-2.200571	1.265479
C	-1.488552	-3.018396	0.112946
H	2.225927	-2.850102	-0.683518
H	-2.225927	-2.850102	-0.683518
H	2.209470	-1.386876	1.372250
H	-2.209470	-1.386876	1.372250

H	-2.891642	-2.145618	1.577802
H	-4.294667	-1.467989	0.582664
H	-3.786409	0.850648	1.443490
H	-2.383954	0.170807	2.439845
C	-2.995410	1.063574	-1.191329
C	-3.314710	-0.231554	-1.658061
H	-3.709091	1.623976	-0.572616
H	-2.270134	1.683203	-1.734844
H	-2.843549	-0.627652	-2.567838
H	-4.278142	-0.691436	-1.401708

1c *cis*

Rh	1.694708	-0.266648	-0.145011
C	2.375984	-0.281733	-2.224011
C	2.628652	-1.917888	0.830116
C	1.564641	-1.410796	-1.995988
Cl	-0.448305	-1.606121	0.819349
H	1.947782	0.619607	-2.679345
C	3.640971	-1.113316	0.283017
H	3.464669	-0.372059	-2.315223
C	2.599197	1.607187	0.780248
H	2.020054	-2.404689	-1.891749
H	2.381755	-1.867203	1.897087
H	0.509128	-1.404398	-2.290253
H	3.674936	1.394863	0.786610
H	2.298838	-2.834184	0.326884
C	1.809562	0.957318	1.748944
H	4.149523	-1.418946	-0.638664
H	4.233798	-0.461298	0.934794
H	2.349290	0.292321	2.439908
C	0.506978	1.480093	2.301815
H	0.704800	2.140448	3.170822
H	-0.065539	2.055778	1.559166
H	-0.133984	0.651980	2.646735
Cl	-0.172188	1.112607	-1.067653

TSbc *cis*

Rh	1.656680	-0.423421	-0.156445
C	2.651331	-0.382785	-1.997873
C	2.537612	-2.124774	0.775998
C	1.751635	-1.497291	-1.955694
Cl	-0.425592	-1.220558	1.300256
H	2.362369	0.531967	-2.534722
C	3.495536	-1.096269	0.709722
H	3.735826	-0.554831	-1.960990
C	2.555659	1.861657	0.760744
H	2.154490	-2.516186	-1.876478
H	1.977222	-2.299445	1.702032
H	0.774937	-1.435375	-2.452956
H	3.584472	1.484084	0.799364
H	2.580803	-2.984622	0.097213
C	1.689970	1.459523	1.745759
H	4.309744	-1.136945	-0.024532
H	3.718579	-0.503306	1.605472
H	2.070809	0.755450	2.499531
C	0.345222	2.059558	2.041089
H	0.448381	2.849269	2.812888
H	-0.117328	2.509953	1.149024
H	-0.347370	1.297160	2.432159
Cl	-0.143046	0.900118	-1.253700
Rh	-2.072413	-0.069182	-0.067520
C	2.329452	2.972620	-0.225716
H	2.766253	2.726251	-1.209443
H	1.263696	3.201031	-0.374113
H	2.835454	3.892876	0.130713
C	-3.507317	-1.507620	0.507414
C	-3.525493	-0.431123	1.423588
H	-2.954463	-2.426763	0.743266
H	-4.317652	-1.630092	-0.222858
H	-4.350578	0.293979	1.408330
H	-2.995915	-0.510383	2.382526
Rh	-2.088322	-0.057035	-0.066653
C	2.266858	2.908075	0.099141
H	2.647780	2.927199	-0.937696
H	1.189103	3.120179	0.069803
H	2.769042	3.734032	0.644050
C	-3.594993	-1.504999	0.245715
C	-3.461415	-0.702978	1.402361
H	-3.111161	-2.489606	0.195421
H	-4.459958	-1.379863	-0.418943
H	-4.220926	0.051639	1.645714
H	-2.870856	-1.058990	2.257208
C	-3.238450	1.708821	-0.303788
C	-3.359380	0.910137	-1.462210
H	-4.071942	1.783766	0.406876
H	-2.536413	2.553571	-0.280847
H	-2.753210	1.124077	-2.353192
H	-4.287159	0.357738	-1.661290

1d *cis*

Rh	1.122988	-0.109845	-0.531076
C	3.231465	2.530222	1.207818
C	1.157896	-0.155210	-2.642155
C	2.217953	2.805881	2.039781
Cl	-0.378362	1.852433	-0.701803
H	3.073213	1.903054	0.319155
C	2.242824	0.620474	-2.170347
H	4.248771	2.910288	1.376917
C	2.815898	-1.284670	0.058326
H	2.358757	3.429598	2.933653
H	1.324117	-1.156171	-3.060067
H	1.210761	2.412239	1.854548
H	3.659597	-0.945522	-0.560312
H	0.239154	0.338326	-2.983800
C	1.864241	-2.116699	-0.591602
H	2.178667	1.716814	-2.159333

C	-3.268383	1.519801	-0.792149
C	-3.307901	0.447168	-1.710724
H	-4.134480	1.736340	-0.152283
H	-2.598576	2.373990	-0.962829
H	-2.669815	0.456365	-2.604935
H	-4.205991	-0.178939	-1.792335

TScd *cis*

Rh	-1.481590	-0.263456	-0.173044
C	-2.629917	-0.780373	2.184691
C	-2.094200	-1.364826	-1.896893
C	-1.659234	-1.723833	2.194489
Cl	0.546541	-1.950335	-0.186814
H	-2.442700	0.222189	2.583765
C	-2.979493	-1.609832	-0.828119
H	-3.659136	-1.008079	1.882179
C	-2.793286	1.349561	-0.524161
H	-1.845050	-2.751723	1.861558
H	-2.418393	-0.735774	-2.734612
H	-0.650481	-1.500358	2.554509
H	-3.734672	0.951133	-0.936451
H	-1.283867	-2.066224	-2.127566
C	-1.702238	1.428003	-1.459395
H	-2.872708	-2.519255	-0.224438
H	-3.985444	-1.169416	-0.823942
H	-1.918171	1.076878	-2.476705
C	-0.634474	2.493653	-1.464411
H	-0.956843	3.312174	-2.140941
H	-0.438245	2.921276	-0.471459
H	0.317475	2.095913	-1.858347
Cl	0.107022	1.000520	1.305145
Rh	2.009430	-0.030230	0.111024
C	-3.024548	2.347538	0.587374
H	-3.714539	1.942522	1.348043
H	-2.093810	2.646279	1.094245
H	3.264614	0.219403	-2.211423
H	2.084731	-2.355673	-1.642214
C	0.986517	-3.147656	0.071646
H	1.489096	-4.136321	0.045086
H	0.755992	-2.901372	1.118216
H	0.031206	-3.250700	-0.473083
Cl	0.004485	-0.312417	1.685362
Rh	-1.889121	0.155542	0.219537
C	3.136567	-1.290550	1.532199
H	3.359499	-0.273061	1.897400
H	2.324426	-1.703271	2.147874
H	4.044634	-1.906624	1.694786
C	-3.545502	1.109403	-0.694788
C	-3.003159	0.148010	-1.576576
H	-3.292503	2.172322	-0.806436
H	-4.491989	0.911937	-0.174664
H	-3.525346	-0.800736	-1.755984
H	-2.319190	0.457933	-2.377642
C	-2.720691	-1.737240	0.692185
C	-3.206145	-0.765006	1.594931
H	-3.371898	-2.134846	-0.097352
H	-1.890333	-2.393515	0.980898
H	-2.755310	-0.658497	2.590973
H	-4.235964	-0.393983	1.513060

TScd *cis* inverted bent structure

Rh	-1.560291	-0.369549	0.001796
C	-1.173823	-0.340336	2.581233
C	-2.557134	-2.044860	-0.867492
C	-0.177679	-1.192983	2.231871
Cl	0.593827	-1.539885	-1.038588
H	-0.981888	0.735344	2.664461
C	-2.819379	-2.008245	0.516111
H	-2.157078	-0.694985	2.912341
C	-3.250911	0.885260	0.134399

H	-3.496091	3.264598	0.177156
C	3.661516	-1.272317	-0.342091
C	3.164510	-0.686714	-1.528619
H	3.384773	-2.300145	-0.072286
H	4.602388	-0.916835	0.099015
H	3.715234	0.124084	-2.023280
H	2.488193	-1.255455	-2.180888
C	2.914488	1.881765	-0.073345
C	3.319328	1.276615	1.136214
H	3.612715	1.951566	-0.918316
H	2.110149	2.628430	-0.074422
H	2.827755	1.542812	2.082094
H	4.333371	0.870736	1.245902

1e *cis*

Rh	-1.334989	-0.444410	-0.087989
C	-2.502919	-2.187487	0.151412
Cl	0.511954	-1.834591	-0.982667
C	-1.699762	-1.999238	1.300091
C	-2.387289	0.852487	1.244548
H	-3.582672	-1.991635	0.194439
H	-2.745865	0.245438	2.088378
H	-2.195346	-2.892525	-0.632841
C	-3.126573	0.722329	0.036500
H	-0.757425	-2.550868	1.408998
H	-2.147632	-1.661090	2.243344
H	-3.968531	0.016059	0.068369
C	-3.298097	1.788609	-1.017106
H	-4.260002	2.313880	-0.845230
H	-2.490589	2.534891	-1.011839
H	-3.345459	1.346036	-2.027983
Cl	-0.044180	1.351123	-1.236004
Rh	1.741608	0.104613	-0.126692
C	-1.617243	2.069757	1.689869
H	-0.747803	1.774002	2.303589
H	-0.313536	-2.280808	2.216641
H	-3.336868	-1.763148	-1.584817
H	0.827609	-0.825678	1.989110
H	-4.130803	0.279567	0.402583
H	-1.778389	-2.703039	-1.270574
C	-2.842472	0.790759	-1.244332
H	-2.261205	-2.664628	1.194321
H	-3.801700	-1.695252	0.892683
H	-3.443788	0.114981	-1.867246
C	-2.255951	1.916160	-2.061348
H	-1.722116	2.664159	-1.459238
H	-1.553639	1.532098	-2.822130
H	-3.079685	2.427296	-2.601612
Cl	0.094432	1.512499	0.079022
Rh	2.108574	0.131289	-0.137783
C	-3.144142	2.138574	0.971257
H	-3.204402	1.900683	2.047763
H	-2.210736	2.695551	0.796935
H	-3.991548	2.816557	0.739621
C	3.509741	-1.387419	0.315289
C	3.739250	-0.911690	-0.995085
H	2.937081	-2.310948	0.475338
H	4.192260	-1.115872	1.131400
H	4.599889	-0.263197	-1.207310
H	3.348648	-1.461759	-1.861649
C	3.306105	1.876135	-0.037147
C	3.084333	1.384704	1.267982
H	4.285762	1.753962	-0.517981
H	2.701148	2.704462	-0.430993
H	2.303081	1.826356	1.901353
H	3.891115	0.879246	1.814580

TScd *trans* conf. II

Rh	-1.487761	-0.205616	-0.057748
C	-2.478126	-0.991844	2.168389

H	-1.248212	2.672610	0.847805
H	-2.267032	2.707377	2.323898
C	2.605999	-1.088666	1.388729
C	3.434040	-1.115135	0.243995
H	1.903457	-1.910171	1.582470
H	2.900225	-0.514333	2.276702
H	4.377365	-0.552974	0.230866
H	3.390253	-1.960604	-0.455571
C	3.007270	1.793926	-0.201516
C	2.261615	1.825717	0.998294
H	4.071814	1.527157	-0.189461
H	2.690783	2.390841	-1.067647
H	1.365945	2.454129	1.075732
H	2.744479	1.587858	1.955113

TScd *trans*

Rh	1.434489	-0.200000	0.086430
C	2.912890	-0.950852	-1.756755
C	1.909441	-0.806465	2.078540
C	2.066716	-2.010925	-1.577271
Cl	-0.551677	-2.037305	0.366060
H	2.676547	-0.162819	-2.480383
C	3.031815	-1.003558	1.253847
H	3.922786	-0.935460	-1.331202
C	2.280428	1.718905	0.360215
H	2.345032	-2.877213	-0.965923
H	1.892759	0.022157	2.789470
H	1.119997	-2.080146	-2.120781
H	3.084624	1.686962	1.116343
H	1.233751	-1.637595	2.310177
C	0.944761	1.739540	0.885798
H	3.259414	-2.005411	0.871688
H	3.891434	-0.322720	1.306343
H	0.176722	2.093013	0.187698
C	0.571180	2.028557	2.319294
C	-2.262627	-1.004442	-1.889287
C	-1.506834	-1.924605	1.970954
Cl	0.550107	-1.817120	-0.588666
H	-2.268755	-0.065605	2.715708
C	-3.054626	-1.440379	-0.811121
H	-3.527980	-1.190871	1.923818
C	-2.628876	1.569445	0.028206
H	-1.725088	-2.897544	1.515962
H	-2.649455	-0.246439	-2.579258
H	-0.479393	-1.759025	2.307946
H	-2.317372	2.071263	0.959178
H	-1.471747	-1.644928	-2.296591
C	-1.716403	1.665708	-1.078735
H	-2.898013	-2.440665	-0.389334
H	-4.050856	-1.018152	-0.640251
H	-2.169809	1.525348	-2.070262
C	-0.521620	2.588294	-1.135925
H	-0.176618	2.885216	-0.135266
H	0.329058	2.121514	-1.665210
H	-0.801082	3.502654	-1.698562
Cl	0.225076	0.826074	1.458499
Rh	2.077321	-0.035896	0.059684
C	-4.127662	1.544588	-0.159220
H	-4.521661	2.581139	-0.155414
H	-4.424908	1.090942	-1.120687
H	-4.639120	1.003032	0.657239
C	3.682007	-1.213739	-0.658601
C	3.191255	-0.392127	-1.698685
H	3.375371	-2.265984	-0.590503
H	4.638575	-0.978264	-0.173220
H	3.761882	0.484126	-2.033811
H	2.489769	-0.800579	-2.438704
C	3.045499	1.843865	0.238422
C	3.443910	0.999101	1.297510
H	3.737466	2.059958	-0.586256

H	1.395228	1.865219	3.033947
H	0.282915	3.095777	2.403235
H	-0.302611	1.433490	2.639748
Cl	-0.098849	0.473094	-1.792507
Rh	-1.909514	-0.108447	-0.188463
C	2.684208	2.437433	-0.905883
H	3.590706	1.992812	-1.354541
H	1.877526	2.421941	-1.656369
H	2.920859	3.497652	-0.677872
C	-3.546471	-1.018670	0.795753
C	-2.805145	-0.240426	1.714927
H	-3.401761	-2.105777	0.745687
H	-4.520183	-0.663310	0.432677
H	-3.195603	0.720313	2.076178
H	-2.069472	-0.721153	2.373672
C	-2.675854	1.864303	-0.363794
C	-3.278328	1.028658	-1.329339
H	-3.242941	2.190043	0.518507
H	-1.850900	2.528648	-0.653442
H	-2.908700	1.022656	-2.364093
H	-4.316914	0.693621	-1.211685

1e *trans*

Rh	-1.253802	0.370451	-0.164641
C	-1.646893	1.765122	1.382811
Cl	0.551740	1.853967	-0.991083
C	-2.407044	2.096462	0.237785
C	-3.050186	-0.746800	0.078971
H	-2.133065	1.325325	2.259885
H	-3.819024	-0.136275	0.580060
H	-0.707669	2.293287	1.592728
C	-2.055502	-1.301898	0.923383
H	-2.051502	2.872341	-0.453266
H	-3.489552	1.917626	0.212598
H	-1.484016	-2.146742	0.512508
H	2.267207	2.601354	0.397211
H	2.974758	1.087810	2.287140
H	4.444044	0.546338	1.303397

1e *trans* conf. II

Rh	1.301415	-0.315414	-0.099874
C	1.756235	-1.671803	1.460064
Cl	-0.460905	-1.891519	-0.817745
C	2.566369	-1.956767	0.337015
C	2.908634	1.099085	-0.284721
H	2.181649	-1.195513	2.352673
H	2.506709	1.830045	-1.002216
H	0.844201	-2.255811	1.638596
C	2.380296	1.138461	1.030659
H	2.293541	-2.768788	-0.350487
H	3.630853	-1.700306	0.344539
H	2.993714	0.646417	1.802599
C	1.482190	2.224149	1.572272
H	2.087444	2.960890	2.139391
H	0.953238	2.754931	0.765500
H	0.729948	1.811323	2.268522
Cl	-0.083798	1.296463	-1.378297
Rh	-1.793166	0.048278	-0.135796
C	4.291966	0.614593	-0.636396
H	4.948948	1.492159	-0.801838
H	4.749086	0.010703	0.165113
H	4.298503	0.026088	-1.571472
C	-3.427074	-1.217729	0.317849
C	-2.608393	-1.071407	1.460611
H	-3.339552	-2.107526	-0.320045
H	-4.395145	-0.702863	0.262316
H	-2.936713	-0.450692	2.304420
H	-1.870680	-1.843664	1.715765
C	-2.369522	1.821227	0.873284
C	-3.134283	1.674141	-0.305430

C	-2.078291	-1.271753	2.429251
H	-1.071372	-1.094746	2.848948
H	-2.768269	-0.513445	2.834752
H	-2.416868	-2.258695	2.804274
Cl	0.037815	-1.325272	-1.441645
Rh	1.728013	-0.121606	-0.131837
C	-3.501490	-1.353575	-1.228041
H	-3.775346	-0.572993	-1.960945
H	-2.721133	-1.988963	-1.676573
H	-4.404629	-1.976742	-1.061933
C	3.429259	1.046624	0.330067
C	2.540676	1.053764	1.429167
H	3.443022	1.886399	-0.377926
H	4.355621	0.458585	0.367925
H	2.771871	0.474448	2.332729
H	1.860522	1.901359	1.583980
C	2.007886	-1.834388	1.075178
C	2.917514	-1.868587	-0.006230
H	2.356265	-1.596726	2.088695
H	1.075024	-2.411169	1.032395
H	2.701449	-2.476382	-0.895243
H	3.981139	-1.652158	0.158240

TScd *trans* inv. bent

Rh	1.515644	-0.260142	0.106637
C	-0.027701	-0.465212	2.520578
C	2.744800	-1.667680	1.105194
C	0.953802	0.456285	2.639785
Cl	-0.563588	-1.756274	-0.528791
H	-1.015667	-0.198679	2.122556
C	2.617740	-2.045640	-0.247081
H	0.109147	-1.511696	2.818284
C	2.735605	0.744548	-1.316758
H	1.925424	0.218235	3.088572
H	3.680665	-1.232199	1.480288

H	-2.823389	1.634176	1.855230
H	-1.496906	2.485511	0.887479
H	-2.858859	2.220782	-1.217465
H	-4.186378	1.365510	-0.251976

TScd inv. bent conf. II

Rh	1.516460	-0.264088	-0.064958
C	0.089449	-1.393929	2.124478
C	2.852289	-1.881955	0.286149
C	1.076038	-0.576917	2.559227
Cl	-0.585179	-1.362206	-1.213443
H	-0.911945	-1.008504	1.890787
C	2.545175	-1.822733	-1.088647
H	0.237737	-2.472588	1.992000
C	2.989177	1.197224	0.358384
H	2.058357	-0.962030	2.856678
H	3.832059	-1.563865	0.656295
H	0.889832	0.488775	2.734146
H	2.413799	2.016798	0.817970
H	2.336714	-2.608595	0.926802
C	2.848039	1.038942	-1.062560
H	1.777386	-2.480970	-1.511888
H	3.281428	-1.452643	-1.811920
H	3.668110	0.479946	-1.542327
C	2.210277	2.034302	-2.001798
H	1.440761	2.644541	-1.506872
H	2.993223	2.710963	-2.403290
H	1.742728	1.525835	-2.864256
Cl	-0.185173	1.569448	0.247709
Rh	-2.148877	0.139136	-0.104257
C	4.273611	0.901321	1.094538
H	4.088951	0.530639	2.118926
H	4.909082	0.170286	0.565639
H	4.865774	1.833786	1.190268
C	-3.754702	-0.832715	-1.079615

H	0.771238	1.504437	2.380052
H	2.029818	1.458530	-1.766896
H	2.126256	-2.156898	1.868293
C	3.171329	1.042420	0.019899
H	1.892980	-2.813034	-0.544839
H	3.454353	-1.909225	-0.936021
H	4.098009	0.529002	0.330466
C	3.005568	2.399228	0.667268
H	2.051988	2.876933	0.389263
H	3.056445	2.334192	1.768486
H	3.828296	3.070388	0.344224
Cl	-0.168960	1.522446	-0.429742
Rh	-2.143210	0.074500	-0.239601
C	3.590658	0.063680	-2.356817
H	4.046158	0.843493	-3.000270
H	4.421941	-0.518899	-1.924637
H	2.998828	-0.593999	-3.018424
C	-3.719229	-1.222344	-0.791462
C	-3.530338	-1.279797	0.607557
H	-3.285762	-1.993521	-1.442299
H	-4.583366	-0.692167	-1.213414
H	-4.249415	-0.798944	1.283999
H	-2.948274	-2.097700	1.053316
C	-3.184890	1.664194	0.699437
C	-3.383413	1.731397	-0.697134
H	-3.993990	1.324676	1.359523
H	-2.430226	2.299617	1.183026
H	-2.787326	2.419303	-1.312269
H	-4.347335	1.440662	-1.135670

t*rans*-octadiene

C	-2.051404	-0.439139	-0.407115
H	-1.931751	-0.403740	-1.502751
C	-1.520984	0.557800	0.322796
H	-1.623126	0.520829	1.420589
C	-3.487486	-1.481048	0.146893
H	-3.359417	-1.237686	-2.020863
H	-4.641782	-0.194199	-1.186090
H	-4.167798	-1.355961	1.000108
H	-2.878987	-2.395148	0.168471
C	-3.151585	1.158165	1.459877
C	-3.404909	1.811081	0.233485
H	-3.935448	0.557181	1.939917
H	-2.379655	1.539368	2.142379
H	-2.833198	2.705078	-0.050834
H	-4.385211	1.718300	-0.252349

$Rh_2Cl_2Et_2$-Octadiene *cis*

Rh	1.009986	-0.175746	-0.320607
Cl	-0.552371	-2.104786	-0.050667
C	1.200452	1.911641	0.049548
H	0.397112	2.303739	-0.587880
C	2.341723	1.469389	-0.676961
H	2.337825	1.674984	-1.757584
C	2.458278	-1.584956	0.447718
H	1.826865	-2.418762	0.780305
C	2.491187	-0.462119	1.293844
H	1.925763	-0.526483	2.233785
C	3.716835	1.314218	-0.054544
H	4.375063	0.801701	-0.777098
H	4.175351	2.313363	0.100899
C	3.652038	0.528637	1.268124
H	4.599979	-0.020513	1.435140
H	3.560680	1.219479	2.121797
Cl	-0.660572	0.463618	-2.005733
Rh	-1.988353	-0.121333	-0.014579
C	3.495154	-1.993532	-0.564995
H	3.020150	-2.498087	-1.425104
H	4.100812	-1.159249	-0.945968
H	4.183811	-2.730940	-0.101376

C	2.051408	-0.439136	0.407115
H	1.931761	-0.403733	1.502752
C	1.520981	0.557799	-0.322797
H	1.623117	0.520824	-1.420590
C	-0.742987	1.721557	-0.224593
H	-0.792467	1.720478	-1.330813
H	-1.204278	2.672564	0.111671
C	0.742985	1.721557	0.224592
H	1.204276	2.672564	-0.111672
H	0.792465	1.720478	1.330812
C	2.799237	-1.620137	-0.140736
H	2.298960	-2.571359	0.130208
H	2.879929	-1.579193	-1.241662
H	3.824326	-1.680064	0.276289
C	-2.799236	-1.620139	0.140736
H	-3.824327	-1.680060	-0.276283
H	-2.298965	-2.571363	-0.130213
H	-2.879922	-1.579198	1.241663

***cis*-octadiene**

C	-2.110865	0.757603	-0.492096
H	-2.222931	1.707043	-1.039158
C	-1.020995	0.013540	-0.768977
H	-0.308626	0.410028	-1.507345
C	2.513064	0.318190	-0.287873
H	3.374688	0.377944	-0.971351
C	1.894255	-0.874285	-0.175325
H	2.275955	-1.704121	-0.790705
C	-0.624636	-1.299192	-0.150541
H	-1.424749	-1.690678	0.502381
H	-0.481341	-2.051522	-0.953778
C	0.691959	-1.204445	0.669949
H	0.554001	-0.459136	1.473750
H	0.858966	-2.180035	1.167674
C	2.158406	1.599410	0.412086
C	1.065285	2.415079	1.462142
H	0.069162	2.143398	1.854990
H	1.824220	2.044933	2.162757
H	1.122203	3.523572	1.458206
C	-3.333747	-1.066779	1.314195
C	-2.317850	-0.411635	2.049827
H	-3.276419	-2.148129	1.130158
H	-4.346775	-0.644877	1.278210
H	-2.533470	0.519888	2.590037
H	-1.456484	-0.977022	2.429775
C	-2.636314	1.880526	0.202348
C	-3.543345	1.173677	-0.620615
H	-2.912691	2.136849	1.233891
H	-1.877313	2.535397	-0.245521
H	-3.480572	1.257399	-1.714032
H	-4.526900	0.872877	-0.237007

$Rh_2Cl_2Et_2$-Octadiene *trans*

Rh	1.125949	0.013454	-0.367171
Cl	-0.515440	-1.459936	-1.483428
C	2.707755	1.421761	-0.024103
H	3.668476	0.885126	-0.053387
C	1.935062	1.263036	1.155015
H	1.165626	2.018246	1.373311
C	2.540065	-1.531472	0.059627
H	3.502711	-1.070880	0.325712
C	1.506989	-1.504712	1.037852
H	0.719480	-2.265526	0.938786
C	2.413108	0.430801	2.339472
H	3.490425	0.210366	2.229127
H	2.306347	1.004497	3.278045
C	1.614945	-0.877115	2.412163
H	2.063540	-1.591220	3.135173
H	0.589616	-0.667190	2.770930
Cl	-0.485251	1.765451	-1.122461

H	3.009999	1.976050	1.012725
H	1.923815	2.393070	-0.324465
H	1.284362	1.497640	1.075819
C	-3.214169	0.450994	0.481360
H	-4.193892	0.396264	-0.033656
H	-3.063682	-0.499524	1.019850
H	-3.308672	1.256091	1.236763

$Rh_2Cl_2Et_2$-Octadiene *cis* Conf. II

Rh	0.992496	0.062863	-0.419255
Cl	-0.657084	-1.382177	-1.551975
Cl	-0.655942	1.839396	-1.004764
Rh	-2.008428	0.027431	-0.048716
C	-3.502145	-1.462574	0.056618
C	-2.544114	-1.686579	1.072319
H	-3.456265	-2.029176	-0.883378
H	-4.492800	-1.060506	0.304616
H	-2.784066	-1.461134	2.120121
H	-1.757915	-2.439402	0.929038
C	-2.386806	1.216858	1.651655
C	-3.408803	1.426579	0.696378
H	-2.580893	0.626756	2.556958
H	-1.558477	1.932805	1.735523
H	-3.381362	2.305659	0.038605
H	-4.406911	0.996535	0.852041
C	2.381875	1.692692	-0.102732
H	1.808473	2.524603	-0.533886
C	1.894627	1.203098	1.135121
H	1.031094	1.722071	1.577203
C	2.309887	-1.649520	-0.341678
H	1.858308	-2.293847	-1.110326
C	1.538719	-1.525300	0.848185
H	0.626915	-2.137001	0.898986
C	2.690408	0.343073	2.098857
H	3.735450	0.254630	1.761080
Rh	-1.873327	0.041288	-0.083991
C	2.662725	-2.613738	-0.986326
H	3.375474	-3.391022	-0.642037
H	1.694422	-3.097051	-1.191586
H	3.054604	-2.211645	-1.938036
C	2.671355	2.631926	-0.925680
H	1.758186	3.229600	-0.784304
H	3.549753	3.271626	-0.703785
H	2.738224	2.349921	-1.991910
C	-3.370217	-1.435650	0.126065
C	-2.402768	-1.598779	1.144008
H	-3.336184	-2.059042	-0.777772
H	-4.357113	-1.016283	0.360086
H	-2.631715	-1.311030	2.178449
H	-1.616354	-2.357113	1.039012
C	-2.281949	1.333462	1.535034
C	-3.271169	1.503050	0.538231
H	-2.516512	0.800844	2.466311
H	-1.441260	2.036371	1.605170
H	-3.208308	2.340477	-0.169660
H	-4.281060	1.098366	0.686609

H	2.727188	0.823957	3.094457
C	2.032640	-1.044500	2.201858
H	2.719911	-1.784648	2.662381
H	1.152990	-0.975845	2.867660
C	3.812779	-1.621211	-0.449150
H	4.148682	-1.188506	-1.407281
H	4.310132	-1.084801	0.371040
H	4.183041	-2.666507	-0.425986
C	3.791413	1.634137	-0.626410
H	4.448983	0.955158	-0.067562
H	3.808845	1.333737	-1.689309
H	4.234182	2.648983	-0.574872

References

1. C. W Bielawski and R. H. Grubbs, *Macromolecules,* 2001, **34**, 8838.

2. M. A. Golub. *J. Polym. Sci.,* 1957**, 25**, 373-377.

3. S. Mavila, I. Rozenberg and N. G. Lemcoff, *Chem. Sci.*, 2014, **5**, 4196-4203.

4. LDC Milton Roy technical note

5. S. Grimme, *J. Comput. Chem.*, 2006, **27**, 1787-1799.

6. S. Grimme, S. Ehrlich and L. Goerigk, *J. Comput. Chem.*, 2011, **32**, 1456-1465.

7. Y. Zhao and D. Truhlar, *Theor. Chem. Acc.*, 2008, **120**, 215-241.

8. A. V. Marenich, C. J. Cramer and D. G. Truhlar, *J. Phys. Chem. B*, 2009, **113**, 6378-6396.
